# 3D Large‐Scale Subwavelength‐Resolution Sound Sheet Tomography Based on an Active and Programmable Circular Meta‐Array

**DOI:** 10.1002/advs.202520560

**Published:** 2026-04-15

**Authors:** Qiu‐De Zhang, Zi‐Bin Lin, Zhuo Chen, Zhao‐Hui Liu, Jin‐Lian He, Jun‐Jie Song, Long‐Sheng Zeng, Liang Zhou, Ming‐Yue Ding, Yu‐Gui Peng, Ming Yuchi, Xue‐Feng Zhu

**Affiliations:** ^1^ School of Physics and Innovation Institute Huazhong University of Science and Technology Wuhan China; ^2^ School of Mechanical Science and Engineering Huazhong University of Science and Technology Wuhan China; ^3^ Department of Biomedical Engineering Huazhong University of Science and Technology Wuhan China; ^4^ China University of Geosciences Wuhan China; ^5^ Huazhong University of Science and Technology and Provincial‐Ministerial Collaborative Major Science and Technology Infrastructure for High‐end Biomedical Imaging Wuhan China

**Keywords:** meta‐array, musculoskeletal imaging, ultrasound computed tomography

## Abstract

Tomography enables volumetric visualization of internal structures with high fidelity, which has transformed diverse fields of science and engineering. Ultrasound tomography, in particular, provides radiation‐free, real‐time, and low‐cost imaging. Conventional linear and planar array designs typically impose inherent limits on aperture, coverage, and resolution, hindering accurate imaging of deep structures. Here, we present an active and programmable circular meta‐array of 2048 channels integrated with cylindrical acoustic lenses, generating a wide‐angle point‐source radiation in plane and a thin flat sound‐sheet beam out of plane. This configuration delivers omnidirectional homogeneous coverage and dynamic focusing, allowing comprehensive full‐matrix echo acquisition. Based on the circular meta‐array, subwavelength resolution ultrasonic images (∼0.8λ) are obtained with delay multiply and sum reconstruction. Extending to the volumetric tomography, the acoustic system reconstructs complex 3D objects and resolves human soft tissues and musculoskeletal structures. Our work provides a versatile platform for ultrasound tomography and opens an avenue for various advanced applications in biomedical imaging and therapeutic monitoring.

## Graphical abstract

A programmable 2048‐element circular ultrasound array combined with a compact acoustic lens produces a thin “sound sheet” over a large field of view, and records echoes with wide angular diversity across the ring aperture. Coherence‐enhanced beamforming converts full‐matrix data into high‐contrast tomographic slices, delivering near‐diffraction‐limited resolution and rapid 2D acquisition for 3D reconstruction in phantoms and human‐limb imaging.

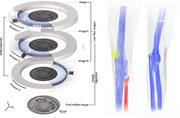

## 1 Introduction

Ultrasound serves widely for diagnostic and therapeutic purposes and remains essential across modern clinical practice due to its non‐invasive, low‐cost, and real‐time feedback without harmful ionizing radiation [1]. Beyond structural imaging of deep organs including the liver, kidney, and thyroid [2, 3, 4, 5, 6, 7, 8], ultrasound also finds broad use in clinical practice, from cardiac function monitoring [9, 10] and real‐time hemodynamic assessment [11, 12, 13] to muscle activity evaluation [14, 15] and volumetric imaging of the skull and bladder [16, 17, 18]. Modern ultrasonic systems are increasingly built on phased‐array front ends that electronically steer and focus beams via element‐level delays and apodization. Within this architecture, synthetic‐aperture ultrasound imaging (SAUI) has emerged as an effective strategy for improving lateral resolution [19]. SAUI interleaves transmissions across array elements and synthesizes an equivalent large aperture through various digital beamforming (e.g., delay‐and‐sum, coherence weighting, or sparsity‐ and learning‐based reconstruction). The framework enhances lateral resolution and sensitivity, sustains rapid acquisition, and enables dynamic focusing across the field of view. Nevertheless, most implementations rely on linear, convex, or gently curved arrays, a geometry that offers limited angular diversity, resulting in information being acquired predominantly from a constrained directional aperture [10, 20, 21, 22, 23, 24, 25, 26]. In addition, the image quality is subject to operator‐dependent variability, including probe pressure and pose, insonation angle, operator proficiency, and patient cooperation [27].

Ultrasound computed tomography (USCT) addresses the tight constraints arising from array geometry and operator dependence through full‐angle insonation, implemented with circular phased arrays, together with physics‐based inversion such as time‐reversal and full‐wave inversion, and thereby enables standardized, quantitative volumetric reconstructions of sound speed and attenuation with improved artifact suppression [28]. Compared with conventional ultrasound imaging, USCT offers a large field of view, reduced image distortion, high spatial resolution, as well as multimodal imaging capability that integrates reflection, transmission, and attenuation contrasts. The circular probes and automated 3D scanning provide complete 360° coverage of the target and comprehensive acquisition of both transmitted and reflected signals, thereby enhancing diagnostic stability and consistency. A major challenge, however, is that the data acquisition and imaging reconstruction are time‐consuming [28, 29], which constrains most current USCT systems to 1024 or 512 channels, or even fewer [30, 31]. Although scan time can be shortened by reducing channel count or adopting uniform undersampling, these strategies inevitably degrade the signal‐to‐noise ratio (SNR) [32, 33] and, due to insufficient spatial and angular sampling, introduce artifacts and erode directional information in reconstructed volumes.

Recent advances in acoustic metasurfaces offer new opportunities for overcoming the limitations that remain in conventional phased‐array and USCT approaches. The acoustic lens, composed of subwavelength structural units, enables the precise manipulation of sound wave propagation [34, 35], including the aberration correction [36, 37, 38], efficient acoustic energy harvesting [39, 40], and super‐resolution imaging [41, 42, 43]. As a compact passive front‐end, the acoustic lens preconditions the transmitted field. Through integration with phased‐array beamforming and USCT reconstruction, it further extends the field of view, enhances the lateral resolution, and improves SNR without increasing the system complexity. With all the pros and cons aforementioned, here we propose an active and programmable circular meta‐array, for which each piezo unit‐cell is integrated with a cylindrical acoustic lens to enable high‐throughput tomographic slice acquisition for volumetric ultrasound reconstruction at subwavelength resolution. Rather than targeting transmission‐mode parameter inversion or a single 2D tomographic image alone, the present system is designed to acquire thin tomographic slices that can be continuously stacked for large‐scale 3D reconstruction. By integrating full‐matrix acquisition with a coherence‐enhanced delay‐multiply‐and‐sum algorithm, the acoustic system achieves concurrent improvements in spatial resolution (∼0.8λ in plane), imaging depth (> 350λ), and acquisition speed (single‐slice acquisition/reconstruction time: ∼0.125 s), while ensuring robustness across heterogeneous targets. The slicing thickness of the sound‐sheet beam is around 1 mm with the ultrasound wavelength of 0.6 mm. Our experimental studies on complex objects and human musculoskeletal tissues demonstrate accurate 3D reconstruction of complicated structural details, establishing a versatile platform with broad applicability in biomedical imaging and clinical diagnostics.

## 2 Results and Discussion

### 2.1 Principles of 3D Ultrasound Tomograph

In conventional ultrasound imaging, data are typically acquired through a sequential scanning approach, where one scan line is transmitted at a time, and the received echoes are subsequently reconstructed. This sequential scheme limits the maximum frame rate due to the two‐way time‐of‐flight across the imaging depth L at sound speed c. The quantitative relationship between the maximum frame rate and the imaging depth L is given by

(1) $$f_{r}=\frac{c}{2LN_{1}}\;$$

where *N*_1_ denotes the number of scan lines per frame. Increasing either *L* or *N*_1_ proportionally lowers *f*_r_, thereby constraining real‐time imaging performance. Furthermore, conventional systems typically employ a single‐focus transmission scheme, which optimizes lateral resolution only at a designated depth. Although segmented focusing can extend the effective focal range, it necessitates multiple transmissions per frame, thereby further reducing the frame rate.

**FIGURE 1:**
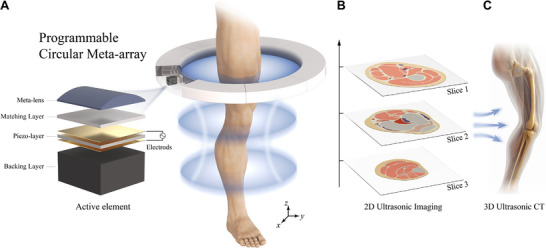
Schematic of 3D tomography imaging based on the active and programmable circular meta‐array. (A) Overview of the programmable circular meta‐array and the structural scheme of a single active element. (B) Three representative 2D ultrasound images of cross‐sectional slices. (C) 3D ultrasonic computed tomography generated from volumetric scanning of multiple slices.

Phased‐array‐based synthetic focusing enables simultaneous transmit and receive focusing throughout the entire imaging depth without compromising temporal performance. Building on this principle, we developed a programmable circular meta‐array system for 3D ultrasonic tomographic imaging (Figure 1A). The system comprises four main components: a circular meta‐array assembly, an excitation and data‐acquisition module, a precision motion‐control unit, and an image‐reconstruction module. Here the circular meta‐array assembly consists of eight concave one‐eighth‐segment array modules, each incorporating 256 independently controlled elements, yielding a total of 2048 elements and forming an annular aperture with an inner diameter of 220 mm. Each active element adopts a multilayer structure comprising a cylindrical acoustic lens, a matching layer, a piezoelectric layer with electrodes, and a backing layer (Figure S1A,B). To balance penetration depth and spatial resolution, the center frequency is set to 2.5 MHz (wavelength: 0.6 mm). The modular annular architecture ensures uniform angular coverage and homogeneous acoustic field distribution across the imaging volume. To provide a fixed and reproducible inter‐module geometry, the eight concave array modules are mechanically constrained by a base plate and locking‐housing assembly, which stabilizes the relative position during handling and operation (Figure S2).

During acquisition, the circular meta‐array is mounted on a high‐precision motorized stage and translated vertically in controlled increments within a water tank, enabling layer‐by‐layer echo collection over multiple cross‐sections (Figure S3). The received signals are transmitted via coaxial cables to a high‐speed acquisition system and subsequently streamed to a server‐based reconstruction platform over a 10‐gigabit ethernet link for later image reconstruction. Once acquisition for a layer is complete, the array advances to the next vertical position until the entire volume is scanned (Figure 1B). The resulting sequence of 2D slices is then rendered into a volumetric dataset to produce the final 3D tomographic image (Figure 1C). The system supports rapid 2D ultrasound imaging and high‐fidelity 3D tomography of arbitrarily complex targets, including acoustically opaque solids, human soft tissue, and musculoskeletal structures, thereby offering broad applicability across a wide range of imaging applications.

### 2.2 Characterization of a Single Active Meta‐Element

In the present tomography scheme, data acquisition is performed by utilizing a transmit‐one, receive‐many configuration, providing comprehensive acoustic field coverage within the horizontal plane (or in‐plane) and employing slice‐by‐slice scanning in the vertical plane (or out‐of‐plane) to construct a 3D tomographic image. Under our operating conditions, a single element provides sufficient penetration and echo SNR for reliable acquisition. When a higher SNR is required, coded excitation can be incorporated to improve SNR without altering the acquisition configuration. For optimal performance, the transmitted wave should approximate a wide‐angle point source in the horizontal plane to maximize lateral coverage, and meanwhile form a sound‐sheet beam in the vertical plane to minimize slice thickness, thereby enhancing synthetic‐aperture tomography by enlarging the angular content of object scattering while preserving sectioning resolution.

**FIGURE 2:**
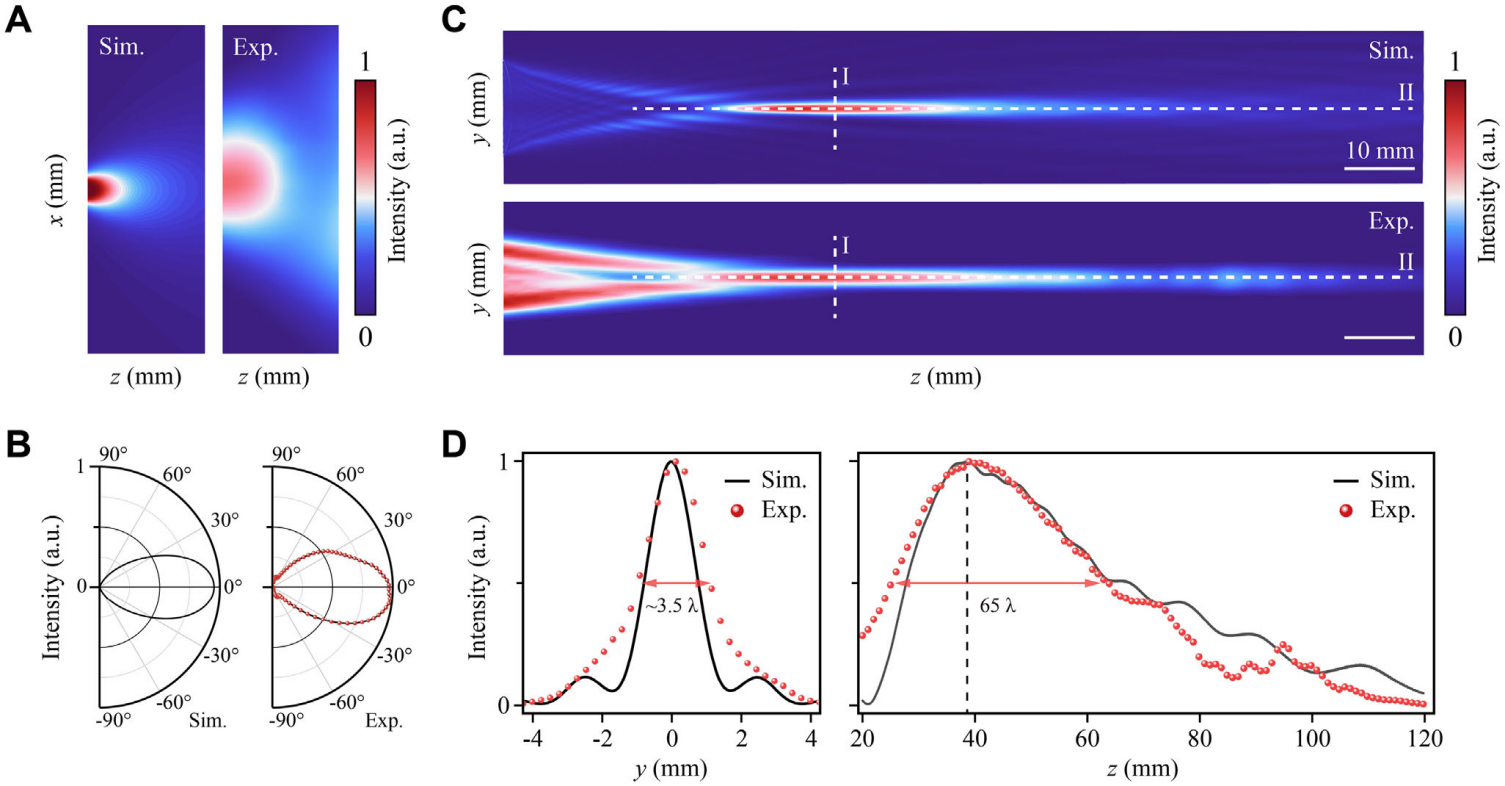
Characterization of the acoustic field from a single active meta‐element. (A) Simulated and measured normalized acoustic intensity distribution in the horizontal plane. (B) Polar beam pattern (divergence angle) for a single element excitation in the horizontal plane. (C) Simulated and measured normalized intensity distributions in the vertical plane. (D) Intensity profiles along the dashed lines I and II in (C) with FWHM of ∼3.5λ and FWHL of 65λ.

To characterize the acoustic field of the one‐eighth‐segment array (256 elements), we use an external field programmable gate array (FPGA) to drive single‐element emissions from the central element of the one‐eighth segment. Simulation and measurement consistently indicate that the field distribution in *x*‐*z* (horizontal) plane resembles a point‐like source with a broad divergence of 80° (Figure 2A,B). In the *y*‐*z* (vertical) plane, the normalized intensity exhibits a sound‐sheet beam with a thin slice thickness and extended axial reach (Figure 2C). This beam shaping is achieved by integrating a cylindrical acoustic lens on the top layer of the one‐eighth meta‐array, which refractively focuses the acoustic waves along the *z*‐axis, thereby reducing slice thickness and improving slicing resolution. The measured full width at half maximum (FWHM, line I), corresponding to the slice thickness, is approximately 3.5λ, while the full width at half length (FWHL, line II) along the beam axis is 65λ (Figure 2D). Notably, although the FWHL peak occurs at 65λ (or 39 mm), imaging is not confined to this position. The acoustic field, as well as the echo signals, extends across the entire horizontal plane inside the meta‐array, with the SNR peak reaching the maximum at 65λ.

### 2.3 USCT Algorithms and System Architecture

Following the acquisition stage, the reflected echoes are processed with delay‐and‐sum (DAS), a conventional beamforming strategy in synthetic‐aperture imaging. The method digitally focuses the RF data by applying the element‐dependent delays to each receive channel and coherently summing the phase‐aligned waveforms to yield the response at the target point. Here, we set the imaging point to be $r_{p}\in R^{3}$, the position of the *i*‐th transmit element $r_{i}$, and the position of the *j*‐th receive element $r_{j}$. The two‐way time of flight (TOF) along the transmit‐receive path is defined by the following equation

(2) $$t_{p}\left(i,j\right)=\frac{d\left(r_{p},r_{i}\right)+d\left(r_{p},r_{j}\right)}{c}=\frac{\left|r_{p}-r_{i}\right|+\left|r_{p}-r_{j}\right|}{c}\;$$

where *c* denotes the sound speed in the medium. The time‐domain RF signal emitted by the *i*‐th transmit element and received by the *j*‐th element is denoted as *S*(*t_p_* (*i*,*j*), *i*, *j*). For any imaging point *p*, the focused amplitude under the DAS scheme is obtained by coherently summing the full‐matrix capture (FMC) dataset from all Tx‐Rx pairs

(3) $$I_{DAS}\left(p\right)=\sum_{i=1}^{N}\sum_{j=1}^{N}w_{i,j}S\left(t_{p}\left(i,j\right),i,j\right)\;$$

where *N* is the total number of array elements and *w*_*i*,*j*_ is the apodization (weight) assigned to each Tx‐Rx pair.

**FIGURE 3:**
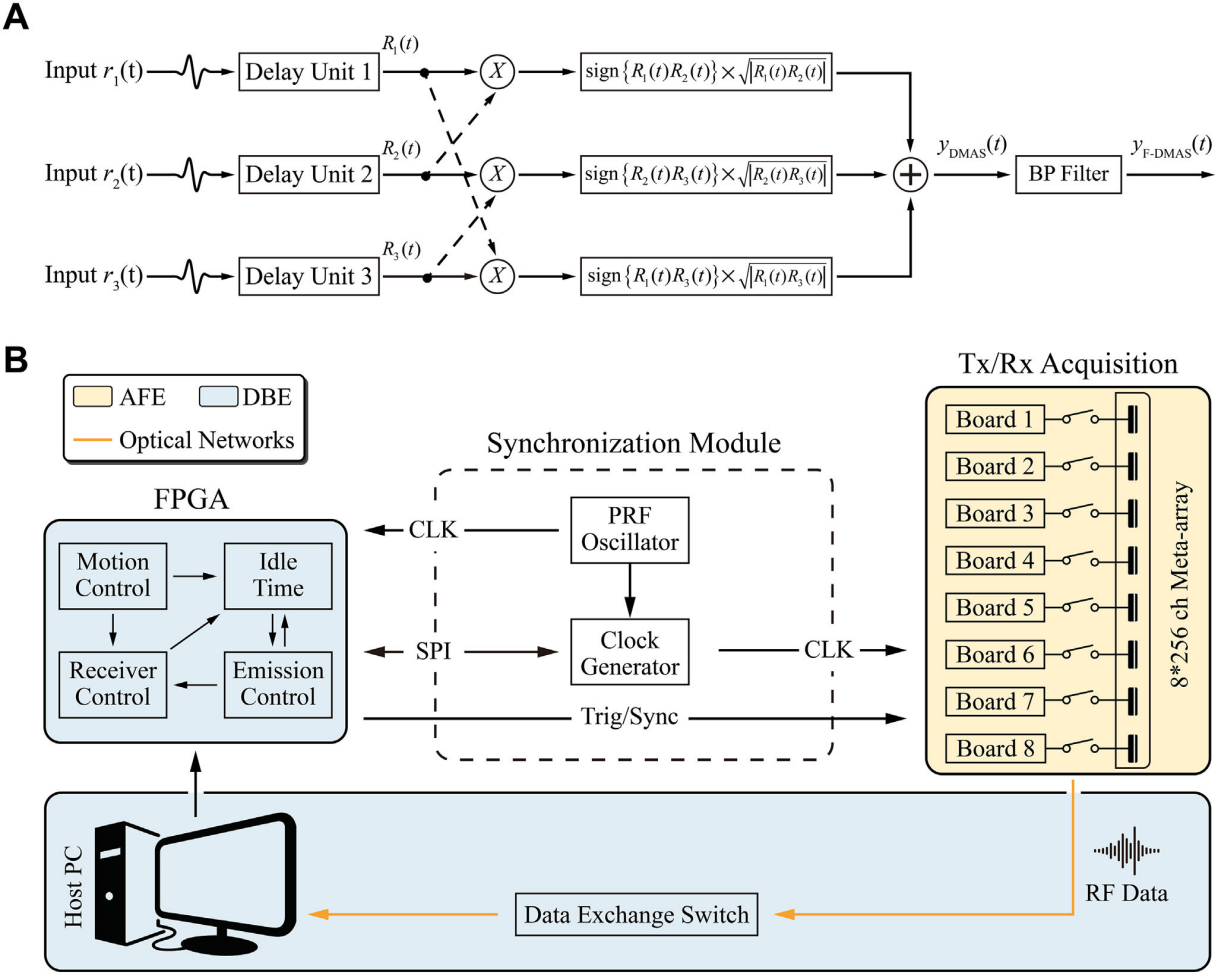
Overview of imaging algorithms and system architecture. (A) Processing flow of the DMAS beamforming algorithm from delay compensation to logarithmic compression. (B) System architecture comprising the active programmable circular meta‐array, Tx/Rx acquisition boards, FPGA, a high‐speed data link, and reconstruction server.

However, despite the computational efficiency and straightforward implementation of DAS, the approach can lead to reduced spatial resolution and a low SNR in the reconstructed images. Here, we apply the delay‐multiply‐and‐sum (DMAS) to highly enhance spatial coherence across the receive aperture by multiplicatively coupling time‐aligned channels [44]. Unlike DAS, which relies on phase‐aligned summation, the DMAS algorithm multiplies time‐aligned channel pairs to exploit inter‐channel correlations and enhance resolution and SNR. Figure 3A displays the processing flow of the DMAS beamforming algorithm. For a single transmit event corresponding to a given scan line, echoes propagating along distinct paths are recorded by the Rx channel. The recorded RF traces are then phase‐synchronized according to the calculated geometric delays (see Equation 2). Following the delay compensation, the signals are coupled in unique pairs and multiplied to reinforce the target echo components. A signed square root is then applied to the absolute product to stabilize dynamic range while preserving polarity. The resulting pairwise signals are summed across all combinations and passes through a band‐pass filter to suppress direct current (DC) and high‐frequency components introduced by the multiplicative operation. Subsequent processing includes demodulation, normalization, and logarithmic compression, ultimately yielding scan lines for image reconstruction. The synthesized signal using the DMAS beamforming algorithm can be expressed as following equation

(4) $$y_{\mathrm{DMAS}}\left(t\right)=\sum_{i=1}^{N-1}\sum_{j=i+1}^{N}R_{i}\left(t\right)R_{j}\left(t\right)\;$$

The sign function quantitatively evaluates phase and waveform consistency between any two received echo signals. After time‐delay compensation is applied to each Rx channel, a value of +1 indicates aligned phases and synchronized waveforms, thereby reinforcing contributions of corresponding signals to the reconstructed image. In contrast, values of ‐1 or 0 denote incoherence, which may introduce artifacts or reduce the SNR. The process produces an equivalent RF signal, which can be expressed as

(5) $${\hat{R}}_{ij}\left(t\right)=\;\mathrm{sign}\left(R_{i}\left(t\right)R_{j}\left(t\right)\right)\cdot \sqrt{\left|R_{i}\left(t\right)R_{j}\left(t\right)\right|}$$

Here, we use *m* to index all the Tx‐Rx couplings of *i* and *j*. The beamformed signal for the DMAS algorithm can then be written as

(6) $${y^{*}}_{\mathrm{DMAS}}\left(t\right)=\sum_{i=1}^{N-1}\sum_{j=i+1}^{N}{\hat{R}}_{\mathrm{ij}}\left(t\right)=\sum_{m=1}^{\left(N^{2}-N\right)/2}{\hat{R}}_{m}\left(t\right)\;$$

Spectral analysis shows that the output of DAS beamforming retains a profile closely matching that of the original RF data, where DMAS beamforming introduces a DC component and a second‐harmonic term due to multiplicative signal coupling (Figure S5). The combined multiplication and summation in DMAS are mathematically equivalent to computing a spatial autocorrelation function. Consequently, the DMAS algorithm can be regarded as an adaptive, correlation‐weighted beamforming method, in which the measured correlation between echo signals received from different directions and positions of the target serves as a weighting factor, thereby increasing the spatial resolution and the SNR.

Figure 3B illustrates the hardware architecture of the USCT platform, comprising an analogue front end (AFE) and a digital back end (DBE). The AFE consists of eight Tx/Rx acquisition boards (Notes S1–S3 and Figures S4–S6), each controlling 256 channels to collectively drive the 2048‐channel circular meta‐array, providing high‐voltage signal excitation, receiving low‐voltage echoes, and performing initial digitization. The DBE comprises an FPGA‐based control unit, a synchronization module incorporating a pulse repetition frequency oscillator and a clock generator for distributing global CLK and Trig/Sync signals, an RF data‐exchange switch connected via optical networks. The host PC performs beamforming, image reconstruction, and post‐processing. Within the control unit, the FPGA coordinates pulse emission, echo reception, motion control, and system synchronization, while the configuration and status monitoring are managed via RS232 communication. Further implementation details of the firmware‐defined programmability, including programmable excitation, real‐time digital conditioning, and buffered high‐throughput streaming, are provided in Note S4.

### 2.4 Fundamentals of Synthetic Aperture Imaging

**FIGURE 4:**
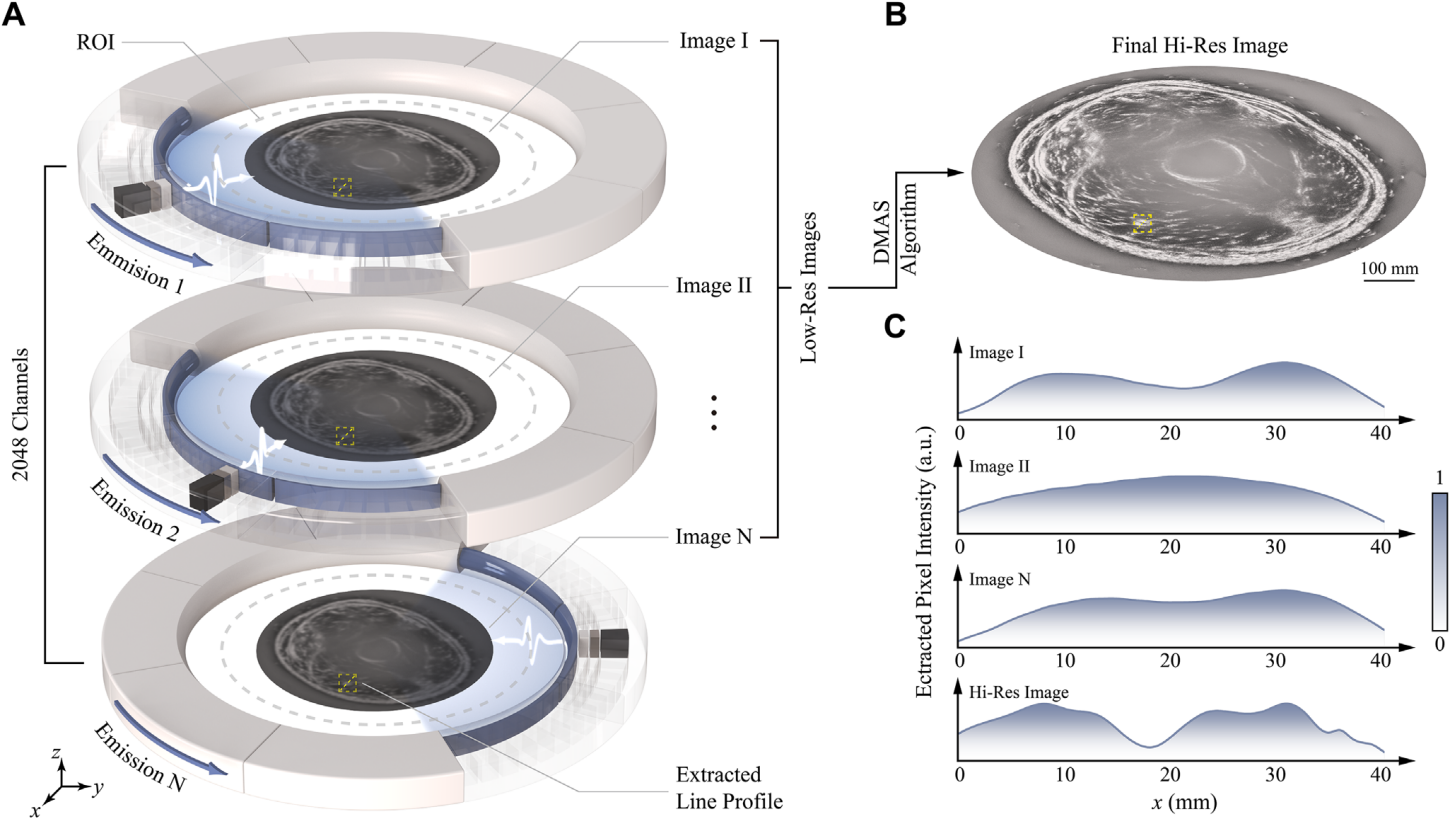
Fundamentals of synthetic aperture ultrasound imaging. (A) Sequential activation of the 2048 array elements with single‐transmit all‐receive acquisitions, yielding multiple low‐resolution images. (B) A high‐resolution image reconstructed via the DMAS beamforming algorithm. (C) Normalized pixel intensity line profiles obtained along corresponding dashed positions in the low‐resolution images in A and in the DMAS reconstruction in B.

The working principle of synthetic aperture imaging with the programmable circular meta‐array is illustrated in Figure 4A. In each firing sequence, only one active element transmits, and a surrounding group of 2048 elements records the echoes, capturing angularly diverse scattering information around the target. As described above, the sequential single‐transmit acquisition follows an FMC protocol and preserves complete transmit‐receive pair measurements for subsequent beamforming and tomographic reconstruction. After appropriate time‐delay compensation, the acquired RF data are utilized to reconstruct a single low‐resolution frame. Sequentially cycling the transmitter across all 2048 elements yields a series of frames from Image I to Image 2048, each carrying complementary angular information yet remaining resolution‐limited before coherent synthesis. Subsequently, we employ the DMAS algorithm to coherently align and combine all intermediate low‐resolution frames, producing a high‐resolution composite image of a transverse section of human thigh (Figure 4B). The elliptical outline marks the region of interest (ROI) within which imaging and analysis were performed. Furthermore, we extracted the line profile from Images I, II, N, and the high‐resolution image (or Hi‐res Image), depicting the pixel intensity along the selected dashed line. Compared with the three low‐resolution images, we observe two distinct intensity peaks at approximately 10 and 30 mm in the high‐resolution line profile, whereas the intermediate frames remain unresolved (Figure 4C). Such image resolution enhancement results from coherently combining all low‐resolution frames into a single composite one, which is effectively equivalent to pixel‐wise dynamic focusing for both transmit and receive (see Methods).

### 2.5 Characterization of 2D Phantom and 3D Complex Object

**FIGURE 5:**
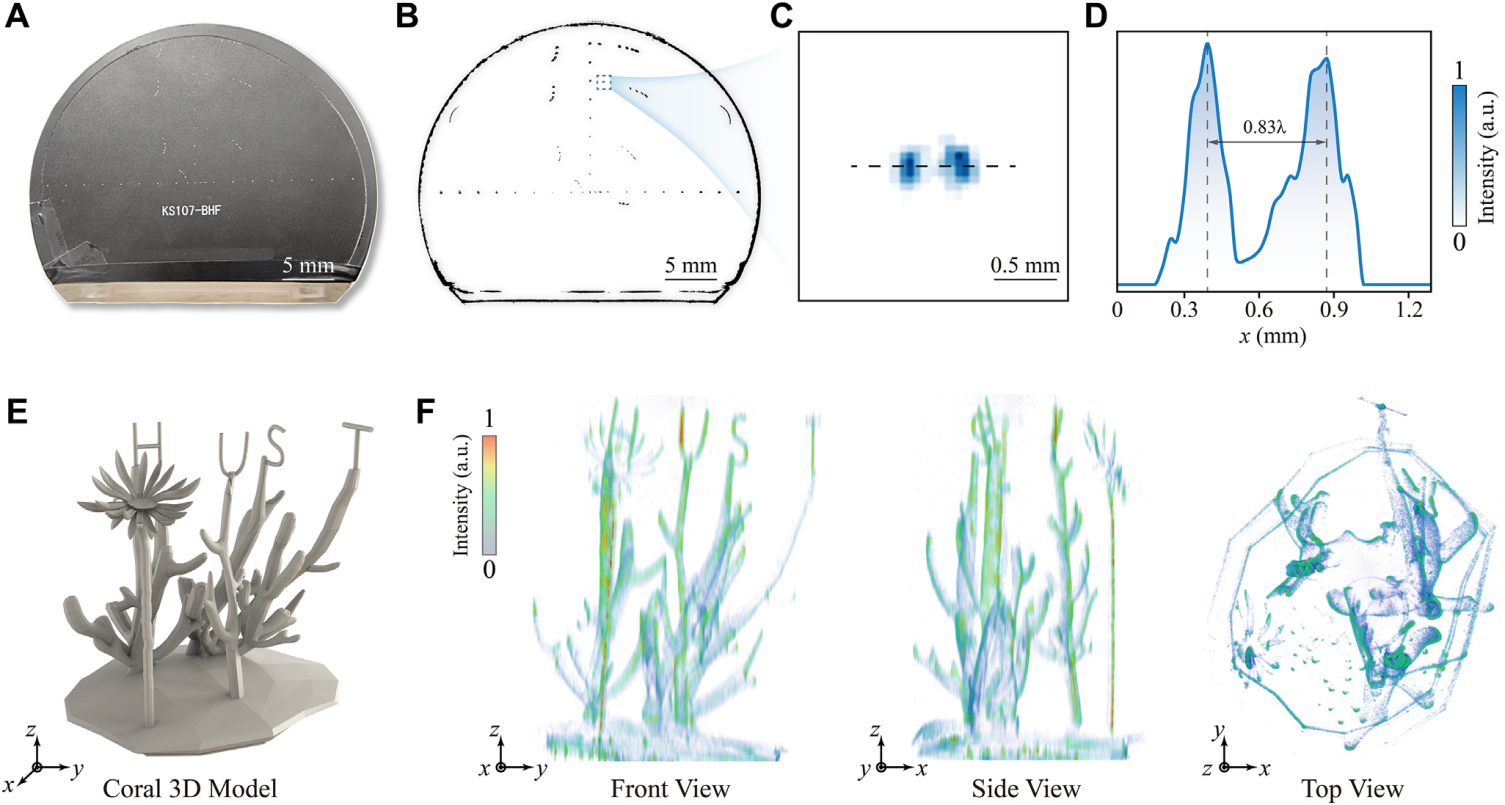
3D tomography imaging for 2D phantom and 3D‐printed object. (A,B), Optical image and tomographic reconstruction of the phantom. (C) Enlarged reconstruction of the boxed region in B. (D) Normalized intensity profile along the black dashed line in C, showing a peak‐to‐peak spacing of 0.83λ. (E) 3D model of a coral specimen. (F) Tomographic reconstruction of the coral from front, side, and top views.

To assess the spatial resolution limit of the meta‐array system, we employed a commercial tissue‐mimicking (TM) phantom (KS107‐BHF, Yice, China), which incorporates clusters of point‐like scatterers at prescribed separations (Figure 5A). Using the 2048‐channel meta‐array in conjunction with DMAS beamforming, we can obtain the cross‐sectional reconstruction shown in Figure 5B. For resolution quantification, a 2 mm × 2 mm ROI containing two adjacent scatterers was selected from the reconstruction (Figure 5C). The lateral intensity profile extracted along the dashed line shows two distinct peaks separated by an intensity dip, corresponding to a minimum resolvable lateral spacing of 0.83λ (Figure 5D), close to the Rayleigh diffraction limit (0.61λ). Notably, the DMAS‐based meta‐array allows acquisition and reconstruction of a high‐resolution 2D image in 0.125 s. In addition, to evaluate the sensitivity of the TM‐phantom reconstruction to inter‐array calibration, we compared the DMAS images reconstructed with and without the calibration. The two reconstructions are nearly indistinguishable, as quantified by a Peak signal‐to‐noise ratio (PSNR) of ∼40 dB (Note S5)

In contrast, a single‐element transducer equipped with a Fresnel zone plate and mechanical scanning can match spatial resolution but requires scanning times that scale exponentially with the target size, making it unsuitable for imaging large objects. Moreover, in conventional mechanical scanning, the lateral resolution will degrade progressively as the imaging plane moves away from the focal point of the meta‐lens, further limiting its capability to imaging large or deep targets (Figure S11).

Following the preceding demonstration of rapid 2D ultrasound imaging, we assessed the capability of the system for 3D tomography of acoustically opaque, high‐impedance‐contrasted targets. Here, a 3D printed coral model, featuring multi‐scale branching and raised alphanumeric details, was selected as the target (Figure 5E). The raised letters “H,” “U,” “S,” and “T” are mounted on slender branches (line width approximately 2 mm), serving as a benchmark for the boundary fidelity and fine‐detail reconstruction. The reconstructed volume faithfully reproduces both the global morphology and the local boundary details of the coral model. Across three orthogonal views (front, side, and top), the slender branches can be continuously resolved, and their intersections are distinctly visible, with the raised letters in the front view being clearly distinguished (Figure 5F). The above findings demonstrate the capability of the system to resolve fine structural and surface details in complex 3D targets.

### 2.6 In Vivo Applications in Medical Ultrasonic Imaging

**FIGURE 6:**
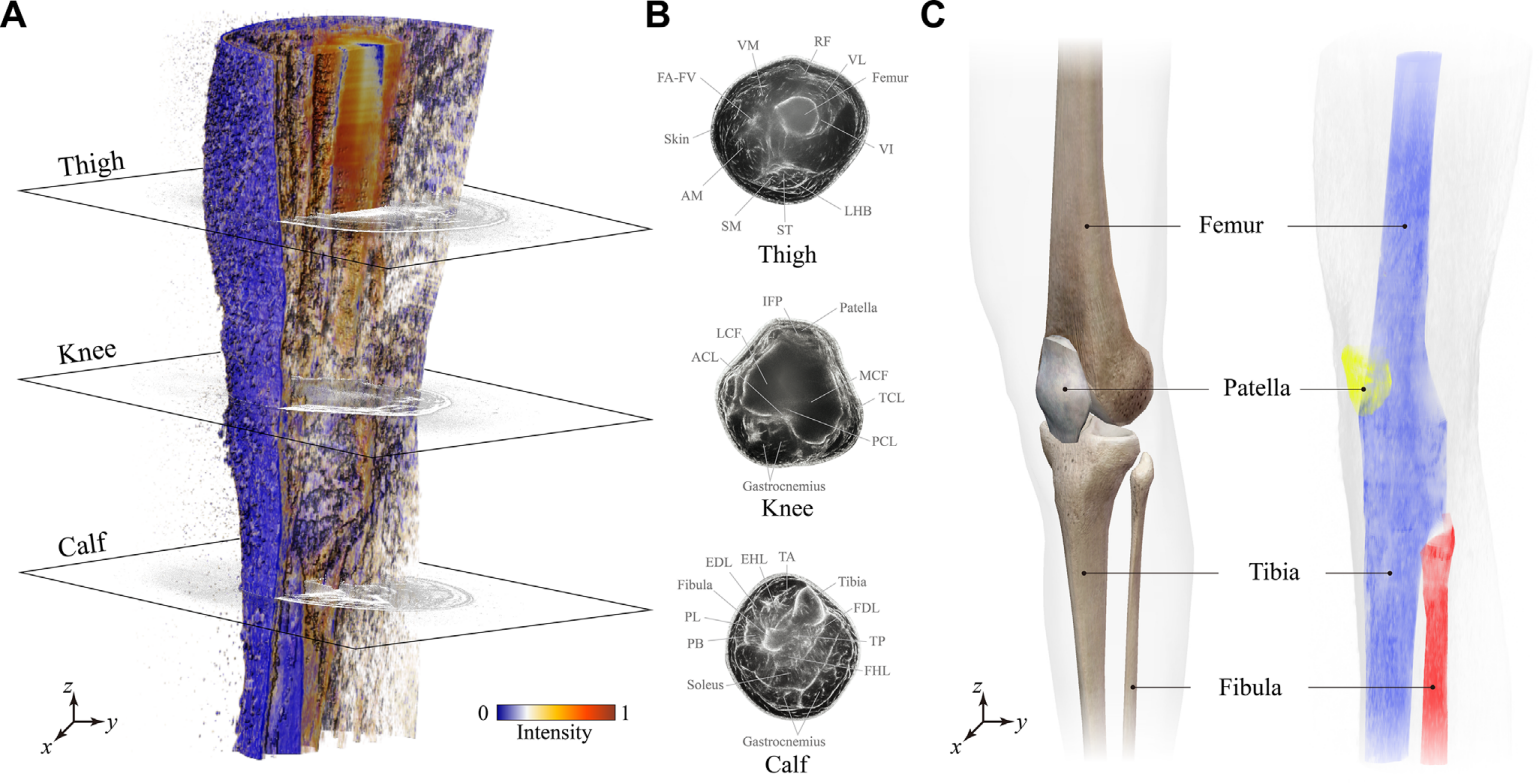
3D tomographic imaging results of human soft tissue and musculoskeletal structures. (A) 3D reconstruction of the right leg spanning the thigh, knee, and calf regions. (B) 2D tomographic slices through the thigh, knee, and calf. (C) An anatomical schematic and the segmented tomographic image of the right leg skeleton.

To validate the applicability of the system in medical diagnostics, we performed in vivo 3D tomographic imaging of human soft tissue and musculoskeletal structures. Both the limb and the circular meta‐array were fully submerged in water to ensure stable acoustic coupling. Prior to immersion, a semi‐oily acoustic coupling agent was applied to the skin surface to establish a gradient impedance transition at the tissue‐water interface. The limb was positioned vertically on a base support to approximate a physiological load‐bearing posture, and the setup was translated along the limb's longitudinal axis to acquire consecutive transverse slices (Figure 1A). The reconstruction was designed to capture the external morphology and contours of the examined regions, as well as internal anatomical features such as bones, major muscle groups, arteries, veins, and nerve bundles. Figure 6A displays the 3D volumetric reconstruction of the human right leg of an adult male derived from all the tomography slices. The rendering spans the distal thigh, knee joint, and proximal calf, providing a continuous anatomical context along the limb. To clearly illustrate the cross‐sectional anatomy, we selected three representative tomography slices of the thigh, knee, and calf regions. These slices delineate the femur, tibia, and the surrounding muscle compartments, providing detailed views of the musculoskeletal structures (Figure 6B). Additional randomly selected slices are provided in Figure S12. Compared to conventional ultrasound and magnetic resonance imaging (MRI), our approach enhances tissue contrast and provides superior visualization of deep tissue structures.

Furthermore, Figure 6C depicts the principal bones from the distal thigh to the proximal calf. The left panel presents a schematic of the overall skeletal distribution, while the right panel shows the segmentation results derived from the tomography volume. The blue region corresponds to the femur (upper) and tibia (lower), while the yellow and red regions represent the patella and fibula, respectively. Segmentation of the 3D tomography data enables precise delineation of individual tissue structures, allowing the designated region rendering through post‐processing (see Methods). In addition, we performed tomography imaging of the upper arm of an adult male to obtain a 3D reconstruction and segmented images of the principal bones (humerus, radius, and ulna) as well as major vessels (Figures S9 and S10).

Compared with 2D ultrasound, 3D tomographic imaging offers a more comprehensive depiction of anatomical structures and more clearly conveys the spatial relationship between adjacent tissues, thereby enhancing the visualization of complex biological structures and facilitating improved diagnostic accuracy. These capabilities are directly applicable to clinical workflows in orthopedics, sports injury evaluation, and postoperative rehabilitation. Serial examinations enable objective assessment of tendon and ligament integrity and facilitate monitoring of inflammatory changes, joint effusion, and other pathological conditions, thus supporting individualized treatment planning and follow‐up.

## 3 Conclusion

In summary, we reported a programmable circular meta‐array system for 3D ultrasound computed tomography, integrating a 2048‐element transducer array, acoustic‐lens‐assisted sound‐sheet generation, and the coherence‐enhanced beamforming. The combination of the circular meta‐array, acoustic meta‐lens, and DMAS reconstruction enables thin, continuously stackable tomographic slices for 3D structural imaging. The system achieves omnidirectional coverage and dynamic focusing across the full imaging volume, enabling high‐resolution reconstructions with rapid acquisition cycles. In the horizontal plane, wide‐angle point‐source‐like emission from single‐element excitation ensures broad coverage and captures scattering information over a wide angular range. In the vertical plane, the acoustic lens generated a confined sound‐sheet beam, which reduces the slice thickness, suppresses out‐of‐plane clutter. Processed with a coherence‐weighted DMAS algorithm, the full‐matrix echoes are converted into voxel‐wise dynamic focusing, effectively integrating angular diversity with spatial precision without compromising coverage. Moreover, this configuration addresses the common limitations of linear arrays, including blind zones, depth‐dependent resolution degradation, and speed constraints of point‐by‐point scanning. Experimental results demonstrate superior performance across high‐impedance TM phantoms and complex object imaging, with distinctive delineation of fine structural and surface details. We further extend the volumetric imaging capability to the biomedical imaging domain, as demonstrated in musculoskeletal diagnostics of the human limbs.

The current implementation reconstructs the elevational dimension through sequential slice acquisition. For dynamic scenarios, high temporal resolution in volumetric tomography is essential for preserving spatial‐temporal consistency and mitigating motion‐induced artifacts, particularly for rapid deformation as encountered in musculoskeletal motion analysis and functional assessment during rehabilitation. Accordingly, future advances toward real‐time volumetric imaging could be achieved by optimizing acquisition strategies and reconstruction schemes, while reducing the number of firings without loss of image resolution. Moreover, mechanically or electrically reconfigurable lenses may also be developed to balance lateral coverage and slice thickness across different imaging scenarios. In addition, to address the data throughput imposed by thousands of channels, hierarchical compression combined with learned‐prior reconstruction is expected to maintain the image fidelity under conditions of limited sampling and reduced SNR ratio. With these developments, the proposed platform has the potential to evolve into a versatile tool for routine musculoskeletal imaging and, more broadly, for rehabilitation monitoring and interventional guidance.

## 4 Methods

All schematic illustrations were generated using Shapr 3D and Cinema 4D 2023 and subsequently rendered in KeyShot Studio. Data analysis was performed using MATLAB R2023a and Origin 2025, whereas finite element simulations were performed in COMSOL Multiphysics 6.3.

### 4.1 Design of the Active Meta‐Element

The programmable circular meta‐array consists of eight one‐eighth segment array modules (Figure S1A), each incorporating 256 independently controlled elements, for a total of 2048 elements. Each element adopts a front‐to‐back layered stack. At the front surface, a molded silicon acoustic lens with an apex thickness of 1.05 mm (*ρ* = 1200 kg/m^3^, *c* = 1000m/s) provides horizontal wide‐angle emission and vertical sound‐sheet confinement. Underlying the acoustic lens, an acoustic matching layer with a thickness of 0.5 mm facilitates impedance matching, reducing reflection and broadening bandwidth. The piezoelectric layer is fitted with top and bottom electrodes and functions to transmit and receive ultrasound waves, with a thickness of 0.525 mm to satisfy the 2.5 MHz resonance requirement. As the base of the element, the 10.5 mm backing layer, consisting of an epoxy matrix loaded with mineral and alumina powders, absorbs all backward‐propagating waves to suppress ringing, thereby improving SNR and forward radiation efficiency (Figure S1B).

### 4.2 Finite Element Simulation

Finite element analysis in the frequency domain was conducted by using the acoustic module of COMSOL Multiphysics 6.3 to characterize the emission field of a single active element. In the simulation setup, the element was simplified as a line segment source emitting plane wave, with the segment length defined by the physical aperture of a single element. Water was set to the propagation medium (*ρ* = 1000 kg/m^3^, *c* = 1500m/s) with radiation boundary conditions applied to the external boundaries. The polar divergence angle and the intensity profiles along the axial and lateral directions were extracted from the horizontal and vertical planes, respectively.

### 4.3 Pressure Field Measurement of the Active Meta‐Element

The transmitting pressure field of a single active element in the programmable meta‐array was measured in a tank with DI water (Figure S4A). Individual elements were selectively excited using an FPGA‐based multi‐channel at a center frequency of 2.5 MHz with a PRF of 1 kHz. The radiated pressure field was captured by a calibrated hydrophone (NH0500, Precision Acoustic Inc., UK) mounted on a 3D scanning system (UMS3, Precision Acoustic Inc., UK). The acquired signals were digitized at a sampling rate of 100 MHz, and the recorded waveforms were Fourier‐transformed to obtain both the spatial amplitude distribution and frequency spectrum of the pressure field (Figure S4B). Echo imaging experiments were performed using a commercial immersion transducer (V384‐SU, Olympus NDT Inc., USA) aligned with a Fresnel zone plate (Note S7 and Figure S11A). The transducer was driven by the pulser/receiver (DPR300, JSR Ultrasonics Inc., USA) equipped with amplifiers for 20 dB gain and an analog band‐pass filter (1∼3 MHz) to suppress the out‐of‐band noise. Echoes from the imaging target at various depths were recorded by an oscilloscope (3034A, Keysight Technologies, Inc., USA) at a sampling rate of 80 MHz.

### 4.4 Dynamic Focusing of Transmitting and Receiving Processes

Dynamic focusing was implemented by pixel‐dependent time‐delay compensation of the RF signals. During receive beamforming, the echo signals *S_i_* (*t*) from each channel was delayed by τ_*i*_, corresponding to the geometric time‐of‐flight in the imaging plane. After delay compensation, the in‐phase signals were coherently summed to yield the focused response (Figure S15). By repeating the operation for each pixel, dynamic focusing was established throughout the entire field of view, leading to signal enhancement at the desired point and suppressing side‐lobe interference. An analogous Tx side synthesis was implemented, in which single‐element firings were sequentially cycled across the meta‐array to construct a synthetic aperture. For each reconstruction pixel, a joint Tx‐Rx delay law was applied so that the effective focus tracked the pixel location rather than remaining fixed at a preset depth, producing approximately uniform resolution and contrast with the slice and a pixel‐wise coherently summed value.

### 4.5 Data Transfer Processing and Image Reconstruction

Raw RF data were acquired with a 2048‐element circular array using 512 hardware transmit‐receive channels at a 25 MHz sampling rate. The minimum acquisition time per elevational slice was 0.125 s, and the maximum generated data volume per slice was 28 GB. The acquisition front end provided high‐voltage excitation and an 80‐tap linear‐phase FIR filter with a 1– 5 MHz passband to suppress out‐of‐band noise. RF frames were continuously streamed over four 10 GbE links to a high‐throughput server at an aggregate bandwidth of about 3.7 GB/s and were written to a 24 TB solid‐state array for subsequent processing.

The reconstruction pipeline comprised RF readout, baseband demodulation with sample‐accurate delay alignment, and DMAS beamforming to generate high‐resolution 2D slices. These operations were executed on an FPGA‐based Data Processing Unit (Xilinx XCVU13P) integrated within the acquisition system. The FPGA adopted a fully pipelined architecture, enabling parallelized delay computation and summation, and achieved up to 8 frames per second for 1024 × 1024 reflection imaging. For visualization purposes, the reconstructed slices were displayed in ImageJ with brightness and contrast (B&C) adjustment, and color inversion was applied, without modifying the underlying reconstructed data.

### 4.6 Standard Resolution Phantom Measurement

Spatial resolution was evaluated using a standard TM phantom, which was configured as a truncated cylinder with a diameter of 180 mm. The phantom contained a TM gel background medium embedding clusters of nylon wires that served as high‐contrast, near‐point scatterers for resolution testing. The material composition and technical specifications conform to the national standard GB10152 and the pharmaceutical industry standard YY/T 0937. The detailed acoustic parameters of the TM phantom are summarized in Table S1. Measurements were carried out with the phantom fully immersed in a tank filled with DI water. Prior to data acquisition, the phantom was soaked for 30 min to reach thermal equilibrium with the surrounding water bath, thereby ensuring stability of the acoustic properties of the TM medium.

### 4.7 Subject Preparation and Acoustic Coupling

The acoustic coupling condition was stabilized by preparing the skin interface to minimize trapped air and bubble‐related reflections. First, the skin surface was cleaned and rinsed to remove surface oils and debris, and then fully wetted to suppress air entrapment within surface microgrooves that may introduce strong reflections. Second, a semi‐oily acoustic coupling agent was applied as a thin and spatially uniform coating on the skin surface. Limited mixing near the tissue‐water interface establishes a graded acoustic‐impedance layer between tissue and the surrounding medium, which suppresses reflections and improves acoustic transmission into the tissue.

### 4.8 Tomographic Rendering and Image Segmentation

Tomographic rendering and image segmentation were performed in Amira (v2024.2, Thermo Fisher Scientific, USA). Consecutive high‐resolution slices were imported as a stacked series to form a 3D volume, with voxel size and bounding box verified during loading to ensure correct physical scaling. Segmentation was conducted using a semi‐automatic workflow guided by anatomical and morphological priors of musculoskeletal structures. Initial label fields were obtained by intensity‐based thresholding, supplemented by interactive painting in regions affected by speckle, partial‐volume mixing, or weak tissue contrast. Thresholding was defined by lower and upper intensity bounds, which were adjusted locally when necessary to preserve inter‐slice continuity of target structures rather than enforcing a single global threshold across the entire volume. Ambiguous boundaries were further refined using marker‐based watershed segmentation. Maker placement was constrained by anatomical plausibility, and watershed separation was computed from gradient‐derived cues, following the standard strategy in which the gradient image was computed and marked regions are expanded to generate connected segmented regions. Following watershed refinement, label fields were post‐processed to improve geometric coherence and suppress isolated artifacts. Small discontinuities were corrected using hole filling and limited morphological adjustment, and the label maps were subsequently smoothed in 2D/3D (shape‐only smoothing) to reduce voxelization effects while preserving thin anatomical features. Isolated components were removed using connectivity‐based filtering, with affected voxels reassigned to the dominant neighboring label. The label fields were then converted into a surface representation for 3D rendering using the Generate Surface module, with conservative smoothing applied only when necessary to mitigate staircase artifacts without distorting fine structures. Segmentation validity was assessed by orthogonal slice inspection, inter‐slice continuity across adjacent slices, and anatomical plausibility of the reconstructed compartments. The resulting segmentations were overlaid on the tomographic volume using distinct colormaps to visualize major structural regions (e.g., skeletal components of the right leg and upper arm), as shown in Figure 6 and Figure S9.

### 4.9 Statistical Analysis

Quantitative data processing was performed using MATLAB and ImageJ, while tomographic rendering and segmentation was conducted in Amira, as detailed in Section 4.8. Imaging results were reconstructed using identical acquisition and reconstruction settings across the compared conditions. For image processing, window‐level settings were adjusted in ImageJ to optimize visualization, and consistent window‐level ranges were applied within each figure panel when making direct visual comparisons. No hypothesis‐testing statistical analysis was performed in this study.

## Funding

This work is supported by the National Key Research and Development Program of China under grant numbers 2020YFA0211400 (X.‐F. Z.) and 2020YFA0211401 (X.‐F. Z.), the Interdisciplinary Research Program of HUST under grant numbers 2025JCYJ001 (X.‐F. Z.), the Fundamental Research Funds for the Central Universities of China under grant number YCJJ20241401 (Z.‐B. L.), and the Natural Science Foundation of Hubei Province under grant number 2025AFD630 (Q.‐D. Z.).

## Conflicts of Interest

The authors declare no conflict of interest.

## Supporting information




**Supporting File**: advs75274‐sup‐0001‐SuppMat.docx.

## Data Availability

The data that support the findings of this study are available from the corresponding author upon reasonable request.
