# Supplementary material for: 3D Large‐Scale Subwavelength‐Resolution Sound Sheet Tomography Based on an Active and Programmable Circular Meta‐Array
Source: Adv Sci (Weinh). 2026 Apr 15;13(39):e20560. doi: 10.1002/advs.202520560 (PMC13335018; doi:10.1002/advs.202520560)
Supplement: Supplementary file 1 — Supporting File: advs75274‐sup‐0001‐SuppMat.docx. [file ADVS-13-e20560-s001.docx]

Supplementary Material for

3D Large-scale Subwavelength-resolution Sound Sheet Tomography based on an Active and Programmable Circular Meta-array

*Qiu-De Zhang, Zi-Bin Lin, Zhuo Chen, Zhao-Hui Liu, Jin-Lian He, Jun-Jie Song, Long-Sheng Zeng, Liang Zhou, Ming-Yue Ding, Yu-Gui Peng*, Ming Yuchi*, Xue-Feng Zhu**

*Corresponding author. Email: [ygpeng@hust.edu.cn](mailto:ygpeng@hust.edu.cn) (Y.-G. Peng), [myuchi@hust.edu.cn](mailto:myuchi@hust.edu.cn) (M. Yuchi), [xfzhu@hust.edu.cn](mailto:xfzhu@hust.edu.cn) (X.-F. Zhu).

**Supplementary Material Guide:**

**Supplementary Notes**

Supplementary Note 1 | High-speed data transmission and acquisition.

Supplementary Note 2 | Tx/Rx acquisition board.

Supplementary Note 3 | Synchronization clock network.

Supplementary Note 4 | FPGA programmability

Supplementary Note 5 | Quantitative evaluation of calibration-induced differences

Supplementary Note 6 | Execution times of 2D tomographic imaging

Supplementary Note 7 | Echo imaging by using the FZP lens.

Supplementary Note 8 | Quantitative analysis of muscle cross-sectional area.

Supplementary Note 9 | Travel-time based delay estimation for beamforming

**Supplementary Figures**

Supplementary Fig. 1 | Schematic of the 1/8 circular meta-array.

Supplementary Fig. 2 | Mechanical assembly and geometric fixation of the Meta-array.

Supplementary Fig. 3 | Illustration of calf scanning and positioning fixation with the developed USCT system.

Supplementary Fig. 4 | The characterization of an active meta-element.

Supplementary Fig. 5 | Architecture of the Tx/Rx acquisition board.

Supplementary Fig. 6 | Photographs of the Tx/Rx acquisition board.

Supplementary Fig. 7 | Time-multiplexed receive scheme

Supplementary Fig. 8 | Architecture of the synchronous clock network.

Supplementary Fig. 9 | Impact of inter-array calibration

Supplementary Fig. 10 | Timing diagram of pipeline tasks.

Supplementary Fig. 11 | Echo imaging of the FZP lens.

Supplementary Fig. 12 | 2D high-resolution ultrasound images of the human leg.

Supplementary Fig. 13 | 3D tomographic imaging of human upper arm.

Supplementary Fig. 14 | 2D high-resolution ultrasound images of the human upper arm.

Supplementary Fig. 15 | Schematic of delay-and-sum beamforming in receive mode

Supplementary Fig. 16 | Comparison of the upper arms between an active and a sedentary volunteer.

**Supplementary Tables**

Supplementary Table 1 | The detailed acoustic parameters of the TM phantom.

Supplementary Table 2 | Comparative analysis of upper-arm tissue cross-sectional areas in two volunteers.

**Supplementary Note 1 | High-speed data transmission and acquisition**

The high-speed parallel transceiver represents the core enabling technology for helical fast imaging in USCT, as its design directly governs the acquisition rate, data throughput, as well as overall system performance. The architecture integrates four principal components: a control backplane, multiple Tx/Rx acquisition board, an Ethernet switch, and a server-based data-offload module that is equipped with an SSD array. The backplane coordinates tasks across boards, distributes low jitter reference clocks and synchronous reset signals, and relays host instructions over RS-232, thereby ensuring unified scheduling and flexible parameter configuration. RD data from each Tx/Rx board are transmitted over 1-Gigabit (1-GbE) uplinks to the switch, aggregated 32 ports, and forwarded over four 10-GbE optical links to a reconstruction server equipped with a 40-Gbps NIC and high-capacity SSDs for real-time storage (see Supplementary Note 2). A clock synchronization generated by the control backplane aligns all acquisition boards to a common timing cycle, ensuring phase-coherent accuracy sampling (see Supplementary Note 3).

A compact state machine embedded in the FPGA firmware governs system operation. After power-up and reset, the system enters an idle mode and awaits an enable command. Upon activation, transmit control is initiated to generate high-voltage pulses, followed by receive control for echo acquisition. This cycle is iterated until all firings across the prescribed slices are completed, after which the motor is halted, and the system returns to idle, awaiting the next command. Through this scheme, synchronized and lossless data acquisition is achieved across 2048 channels, while maintaining scalability and compatibility with standard Ethernet protocols. The resulting architecture provides a robust and upgrade-ready data path that supports continuous high-throughput operation for volumetric USCT.

**Supplementary Note 2 | Tx/Rx acquisition board**

Figures S5 and S6 present the architecture and corresponding photographs of a lab-designed Tx/Rx acquisition board for the ultrasound imaging system, respectively. Serving as the central hardware module of the system, the board integrates multi-channel high-voltage excitation, front-end echo conditioning, and synchronized data acquisition and transmission. It interfaces with multiple piezoelectric array modules through high-density connectors and incorporates high-voltage multiplexing together with T/R switching, enabling programmable transmit and receive configurations across the array. In the receive chain, the echo signals are first conditioned by the analog front-end (AFE) circuitry and then routed to the FPGA control unit. The FPGA coordinates channel selection and acquisition timing and uses FIFO buffering to sustain high-throughput streaming. The acquired data are subsequently transmitted to a host PC via Ethernet for storage and downstream image reconstruction. To support stable operation during multi-channel, high-frequency acquisition, the board further integrates power management and clock synchronization modules, together with multiple gigabit Ethernet interfaces.

A key feature of the board-level interface is the time-multiplexed receive architecture enabled by the 4:1 multiplexing scheme. Specifically, four transducer elements are mapped to a single pulser channel and a single AFE receive channel, resulting in 64 pulser channels and 64 AFE/data-acquisition channels for each 256-element module. Under this configuration, full-aperture receive data are obtained through segmented acquisition. As illustrated in Figure S7, for a given transmitting element, the excitation is repeated four times, and in each firing the receive multiplexer selects a different one-quarter subset of the ring aperture. The four receive subsets are then concatenated to form the full-ring receive dataset associated with that transmit event. This acquisition strategy provides the complete set of measurements required by the FMC-based reconstruction while maintaining a practical front-end channel count.

**Supplementary Note 3 | Synchronization clock network**

As illustrated in Figure S8, a high-precision, low-phase-noise, low-jitter oscillator (OSC) on the back plane serves as the master reference for the FPGA. Subsequently, the FPGA drives a zero-delay clock buffer, which then feeds a dual phase-locked loop (PLL) clock generator that synthesizes the synchronization clocks delivered to each Tx/Rx acquisition board. A backup reference OSC can also be routed to the generator to maintain continuity. When a Tx/Rx board operates in isolation, an on-board precision active OSC provides the local reference. In multi-board operation, however, each board receives the synchronization clock from the control backplane, ensuring a common timing cycle and synchronous echo acquisition. On each board, the FPGA regenerates a zero-delay clock, which passes through a jitter cleaner and is distributed by a clock-distribution integrated circuit to the analog front end (AFE). This arrangement minimizes skew and jitter accumulation, thereby maintaining phase-coherent sampling accuracy and deterministic synchronization across the system.

**Supplementary Note 4 | FPGA programmability**

Building on the system-level data path, acquisition board architecture, and synchronization clock network described in Supplementary Nots 1-3, the imaging platform employs an FPGA-based digital front end in which firmware-defined control logic and software-configurable parameters provide programmability for acquisition and data processing. Within this framwork, programmable functions include excitation sequencing, timing and channel addressing, real-time digital signal conditioning, and buffered high-throughput data streaming, without requiring modification of the analog front-end hardware. Programmable control is implemented via a command-based host-FPGA interface, where structured command frames configure acquisition parameters and sequencing logic. This interface enables software-defined adjustment of excitation timing, channel addressing, trigger order, and data-handling modes without modification of the analog front end, while reserved parameter hooks in the control logic facilitate functional extension through firmware updates.

Digitally programmable excitation is realized by translating software-defined pulse specifications into hardware-timed waveform generator. Configurable parameters include code length, symbol definition, and chip duration, supporting arbitary digital pulse sequences with code lengths up to 4096 symbols and multiple coding families (PLFM, Barker, Golay, and M-sequences) when coded excitation is reqiured. Fine timing control is achieved using a combined coarse-fine delay scheme, with selectable fine delay of 0, 1.25, 2.5, or 3.75 ns. A DDR-based high-rate output strategy (400 MHz, double-edge) provides an effecrive timing resolution of 1.25 ns, and I/O placement contraints are applied to suppress channel-channel skew and preserve deterministic synchronization, with the same approach supports compensation of PCB trace-induced propagation delays on the order of ~50 ps.

Real-time signal conditioning is integrated into the FPGA datapath to maintain continuous acquisition. A representative implementation uses a pipelined multi-channel FIR architecture (*e.g.*, 80 taps across 16 channels), where time-multiplexing maps a 25 MHz input stream to an effective 400 MHz internal processing rate before demultiplexing back to parallel outputs. The processing chain is implemented in fixed-point arithmetic for deterministic latency and resource effciency, with input delays selectable from 1 to 16 clock cycles using shift-register primitives and multiply-accumulate operations executed on dedicated DSP blocks. Filter coefficients are quantized by scaling (by a factor of 2^24^) and rounding to integers prior to loading into the processing pipeline.

To accomodate the data volume in tomographic acquisition, a buffered high-throughput streaming architecture decouples acquisition from back-end transmission. Memory access and network transmission operate in separate clock domains (DDR3 controller at 200 MHz, 10 GbE domain at 145.25 MHz), bridged via asynchronous FIFOs to sustain stable throughput under continuous load. Buffered echo data are encapsulated into UDP packets on the FPGA and streamed to the host through the MAC/PHY interface, with clock integrity maintained by a jitter-cleaning clock multiplier generating the 156.25 MHz reference required for stable 10 GbE transceiver operation. Board-to-board exchange is additionally supported through multi-lane LVDS links, providing aggregate bandwidths up to 9.6 Gbps (under full-duplex operation) while accommodating 8-16 bit data widths for compatibility with different front-end configurations.

**Supplementary Note 5 | Quantitative evaluation of calibration-induced differences**

To quantitative assess the impact of inter-array calibration on tomographic reconstruction, we evaluate the similarity between reconstruction obtained with and without calibration using the peak signal-to-noise ratio (PSNR). The evaluation is performed on the reconstructed image of the TM phantom, and representative reconstructions together with the corresponding relative error map are shown in Figure S9. As shown in Figure S9A and B, the reconstructions with and without calibration are nearly indistinguishable in terms of the TM phantom, and the relative error map exhibits only low-amplitude differences without signatures of global inter-array misalignment. To quantify this observation, let *I*_cali_ and *I*_cali_ denote the reconstructed images with and without calibration, respectively. The mean square error (MSE) is defined as

$MSE =\frac{1}{N}\Sigma_{k=1}^{N}\left( I_{\text{Cali }}\left( k \right)-I_{\text{NoCali }}\left( k \right) \right)^{2}$, (S1)

where *N* is the pixels.

The PSNR is then computed as

$\text{PSNR=10}{log}_{10}\left( \frac{\left( \max\left( I_{\text{Cali}} \right) \right)^{2}}{\text{MSE}} \right)$, (S2)

where *max*(*I*_Cali_) denotes the maximum pixel intensity in the calibrated reconstruction, consistent with common practice for floating-point ultrasound images.

Using this metric, the PSNR between calibrated and uncalibrated reconstructions is approximately 40 dB (39.38 dB), indicating a high level of consistency between the two images. These results confirms that the inter-array geometry is mechanically stable and reproducible, and that calibration introduces only minor refinements.

**Supplementary Note 6 | Execution times of 2D tomographic imaging**

To improve real-time reconstruction performance, a two-stage pipeline processing strategy was implemented, consisting of data acquisition and image reconstruction, as shown in Figure S10. For each 2D tomographic slice, data acquisition requires approximately 21 ms for sampling and 104 ms for front-end to back-end data transfer, yielding a total acquisition time of 0.125 s per slice. In the implemented timing schedule, the sampling stage corresponds to 128 firing envets, each assigned an acoustic sampling window of approximately 164 µs, giving a net sampling time of 164 µs × 128 = 20.992 ms, consistent with the reported ~21 ms. Image reconstruction stage is executed in parallel with data acquisition, where the previous slice is reconstructed while the subsequent slice is being sampled and transferred. The reconstruction time is approximately 108 ms, therefore, the overall throughput is governed by the acquisition stage, resulting in an effective imaging rate of approximately 8 frames per second (fps) for 2D tomographic imaging.

**Supplementary Note 7 | Echo imaging by using the FZP lens.**

The Fresnel zone plate (FZP) focusing lens consists of concentric rings with gradually decreasing widths. The alternating transparent and opaque regions modulate the incident acoustic waves and produce constructive interference at the designed focal point. The radius of the *n*th concentric ring is defined by

$r_{n}=\sqrt{n\lambda F+\frac{n^{2}\lambda}{4}},$ (S3)

where *F* denotes the focal length (20mm in our design), *λ* is the acoustic wavelength, and *r_n_* is the radius of the *n*th ring (*n* = 1, 2, 3, …, *N*). The corresponding ring width is determined by

$w_{n}=r_{n+1}-r_{n}\text{ }\left( n=1,2,3\cdots\right),$ (S4)

where *w*_n_ represents the width of the *n*th ring counted from the center.

The ultrasonic imaging was performed in a tank with DI water (see Figure S11A). The commercial transducer was aligned with the FZP lens, and the imaging object was first positioned at the focal plane (*F* = 20mm). Echo images were obtained by mechanically scanning the object through the 3D stepper (Figure S11B). As the imaging distance increased from 20mm to 60mm (100λ) and 120mm (200λ), the FWHMs of intensity profiles extracted along the dashed lines gradually increased, indicating a progressive degradation in the lateral resolution (Figure S11C).

**Supplementary Note 8 | Quantitative analysis of muscle cross-sectional area.**

Nowadays, muscle health constitutes an essential indicator of physical capacity and overall physiological status. Quantitative assessment of muscle cross-sectional area (CSA) provides a robust measure for evaluating tissue composition and functional status. To this end, USCT was employed to analyze upper-arm CSA, with emphasis on the lifestyle-related differences and the methodological applicability of USCT in muscle health monitoring. Two healthy male volunteers, both 23 years of age, were recruited: volunteer A regularly engaged in physical exercise, whereas volunteer B reported a predominantly sedentary lifestyle. Slices were conducted at the mid-upper arm.

Representative transverse USCT slices are presented in Figure S16A, which corresponds to volunteer A, and Figure S16B to volunteer B. Comparison of the two slices indicates an increased thickness of the subcutaneous adipose layer in the sedentary participant. Tissue compartments, including bone, muscle, subcutaneous fat and coupling gel, were systematically segmented and assigned colors for visualization (Figures S16C and S16D). Quantitative analysis of segmented regions was subsequently performed, and the calculated CSA are summarized in Supplementary Table 2. Although the total CSA of volunteer A’s upper arm was slightly smaller than that of volunteer B, the proportion of muscle tissue was higher in volunteer A (60.98%) than in volunteer B (46.12%). The larger muscle fraction in volunteer A is in accordance with the physiological effects of regular physical exercise, whereas the lower muscle proportion and relatively high fat content in volunteer B reflect morphological features commonly associated with sedentary behavior. These results demonstrate that USCT enables high-fidelity discrimination of tissue compartments and provides quantitative measure of muscle status. The approach offers objective indices for individualized health evaluation and presents potential utility in clinical applications, particularly in the diagnostic assessment and rehabilitation monitoring.

**Supplementary Note 9 | Travel-time based delay estimation for beamforming**

To improve the physical consistency of delay estimation in heterogeneous media, we consider the travel-time field $T\left( \boldsymbol{x} \right)$, defined as the time for an ultrasound wavefront $\sum$ to reach a spatial point x. Let $F\left( \boldsymbol{x} \right)$ denote the local wavefront paropagation speed. The fist-arrival travel time satisfies the Eikonal equation,

$\left| \nabla T\left( \boldsymbol{x} \right) \right|F\left( \boldsymbol{x} \right)=1$, $T\left( \boldsymbol{x} \right)=0,\boldsymbol{x}\in\sum_{0}$, (S5)

where $\sum_{0}$ denotes the initial wavefront.

On a 2D Cartesian grid, a first-order upwind discretization can be used to approximate the spatial gradient magnitude. Denoting the discrete travel time at grid node (*i*, *j*) by $T_{ij}$, and introducing the one-sided finite difference $D_{x}^{-}T_{ij}$, $D_{x}^{+}T_{ij}$, $D_{y}^{-}T_{ij}$, and $D_{y}^{+}T_{ij}$, the upwind Godunov approximation leads to

$\left[ \max\left( D_{x}^{-}T_{ij}, {-D}_{x}^{+}T_{ij}, 0 \right) \right]^{2}+\left[ \max\left( D_{y}^{-}T_{ij}, -D_{y}^{+}T_{ij}, 0 \right) \right]^{2}=\frac{1}{F_{ij}^{2}}$, (S6)

where $F_{ij}$is the local speed at node (*i*, *j*).

If $\nabla T$is approximated using first-order finite differences, Equation S6 can be rewritten in compact form as

$\sum_{\nu=1}^{2}\left[ \max\left( \frac{T-T_{v}}{\Delta_{v}},0 \right) \right]^{2}=\frac{1}{F^{2}}$, (S7)

with the identification $\Delta_{1}=\Delta_{x}$,$\Delta_{2}=\Delta_{y}$,$T=T_{ij}$, and $F=F_{ij}$. The upwind nerighbor times are defined as

$\left\{ \begin{aligned} &T_{1}=\min\left( T_{i-1,j},T_{i+1,j} \right) \\ &T_{2}=\min\left( T_{i,j-1},T_{i,j+1} \right) \end{aligned} \right.$ (S8)

With these definitions, the local update for *T* admits a piecewise solution depending on the relative ordering of *T*_1_ and *T*_2_. If the updated value satisfies *T* > max (*T*_1_, *T*_2_), both coordinate directions contribute to the upwind gradient and *T* is obtained as the physically admissible root of the correspongding quandratic equation implied by

${\sum_{\nu=1}^{2}\left( \frac{T-T_{v}}{\Delta_{v}} \right)}^{2}=\frac{1}{F^{2}}$. (S9)

If only onedirection is active, the update reduces to a 1D step.

Specifically, when *T*_2_ > *T* > *T*_1_, the solution becomes

$T=T_{1}+\frac{\Delta_{1}}{F}$, (S10)

And when *T1 > T > T2*, the solution becomes

$T=T_{2}+\frac{\Delta_{2}}{F}$, (S11)

These local updates constitute the standard upwind travel-time solver that underpins fast marching type computations, emabling the construction of a travel-time over the imaging grid. In conventional reflection-mode USCT, the image is often reconstructed using DAS beamforming under the assumption of a spatially uniform sound speed. The DAS reconstruction can be written as

$R_{\text{DAS}}\left( m,n \right)=\sum_{\alpha=1}^{A}\sum_{k=1}^{K}R\left( k,t-\Delta t_{m,n} \right)W_{m,n}$ (S12)

where *R*_DAS_(*m*, *n*) denotes the beamformed intensity at pixel (*m*, *n*), *R*(*k*, *t*) is the raw echo signal recorded by receiving channel *k* at samplign time t, *K* is the number of adjacent receive channels contributing to the reconstruction, and *A* is the number of transmit events on the circular array. The delay Δ*t_m_*_,_*_n_* is the time-of-flight from the transducer element to the predefined imaging grid point (*m*, *n*), computed using a constant propagation speed, and *W_m,n_* denotes an aperture weighting factor. When a travel-time map Tm,n is computed using the fast marching foumulation above, it can be used to perform sound-speed DAS beamforming by replacing the constant-speed delay mat Δ*t_m_*_,_*_n_* with *T_m,n_*,

$R_{SOS-DAS}\left( m,n \right)=\sum_{\alpha=1}^{A}\sum_{k=1}^{K}R\left( k,t-T_{m,n} \right)W_{m,n}$. (S13)

In this form, the travel-time map *T_m,n_* implicitly accounts for spatial variations in propagation speed and provides a physics-consistent alternative to constant-speed delay estimation.


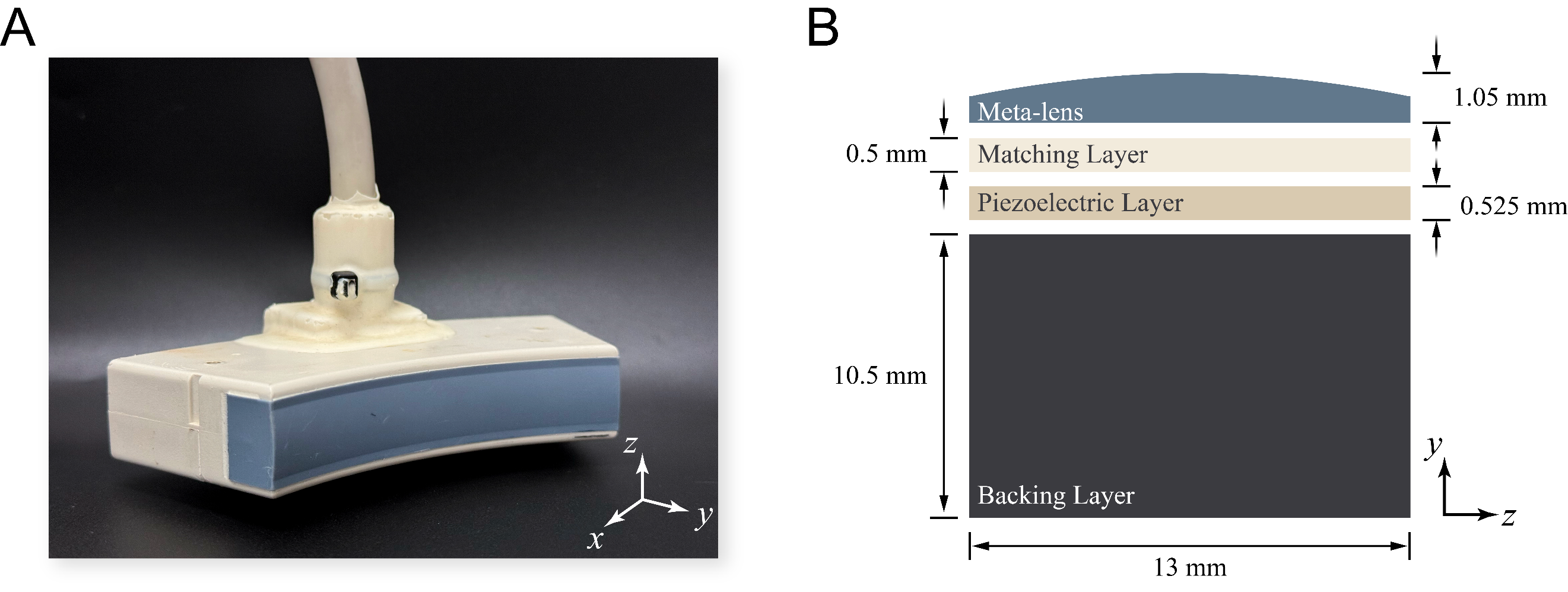


**Fig. S1. Schematic of the 1/8 annular meta-array.** A, Optical photograph of the 1/8 annular array. B, Cross-section dimensions of a single array element.


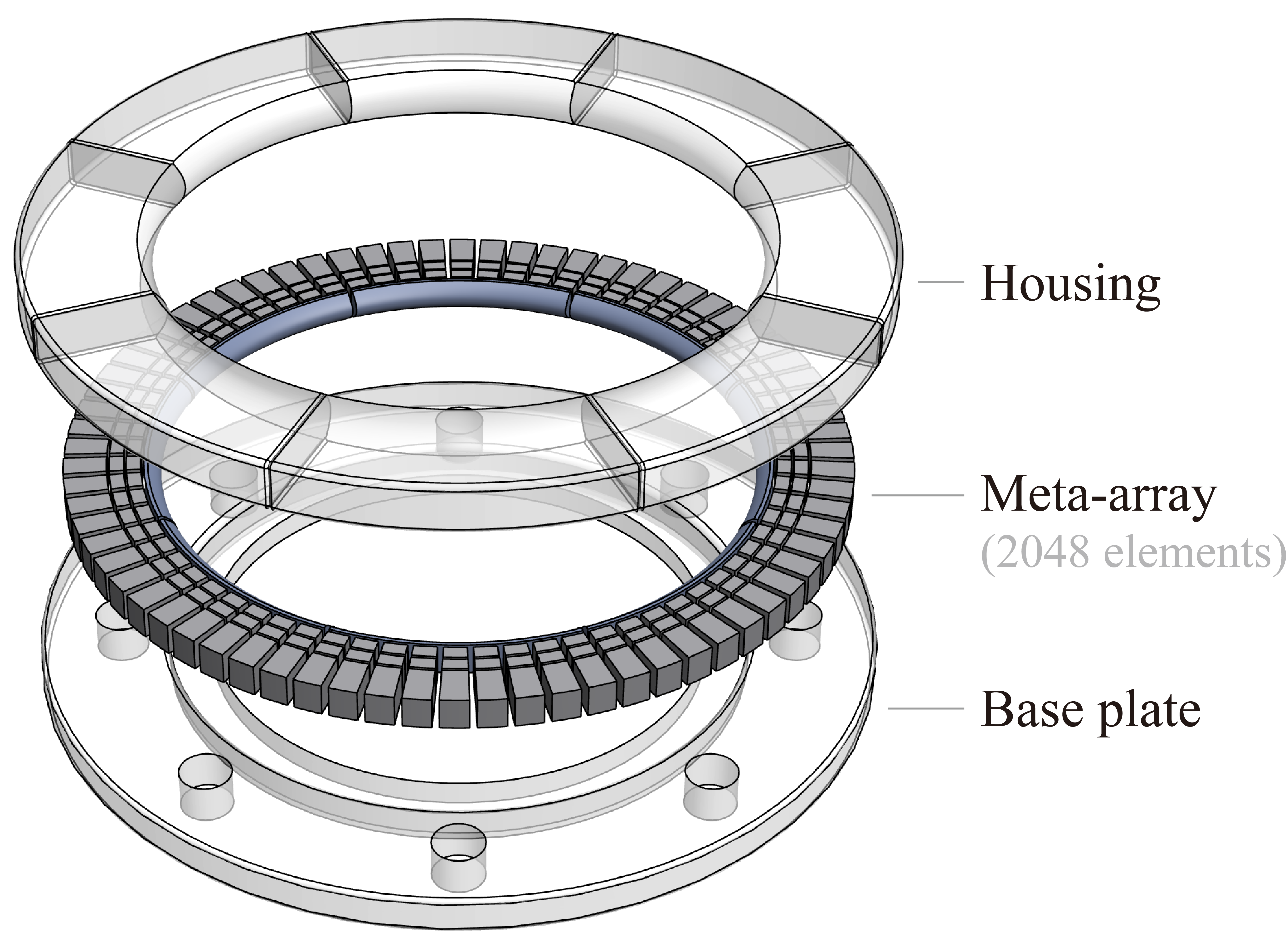


**Fig. S2.** Mechanical assembly and geometric fixation of the Meta-array.


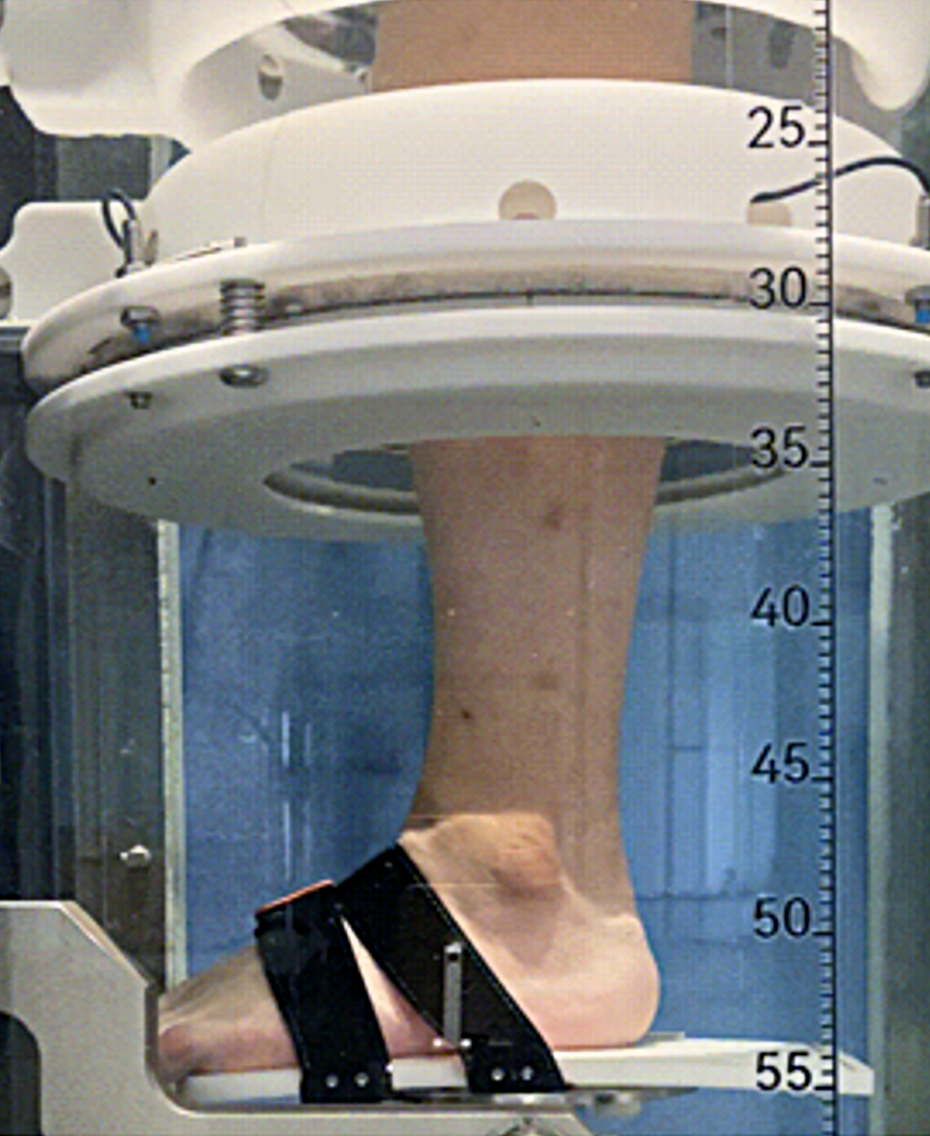


**Fig. S3. Illustration of calf scanning and positioning fixation with the developed USCT system.**


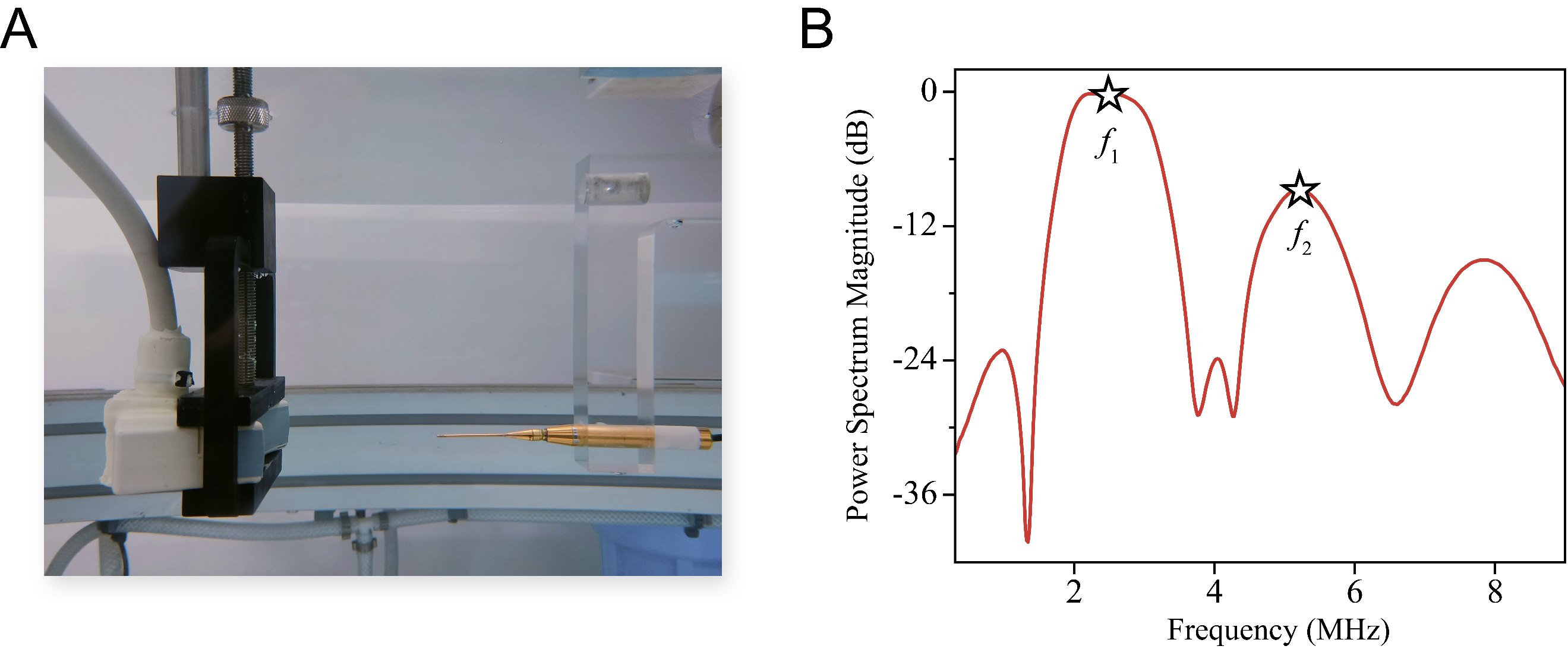


**Fig. S4. The characterization of an active meta-element.** A, Experimental setup. B, Measured frequency spectrum of an active element.


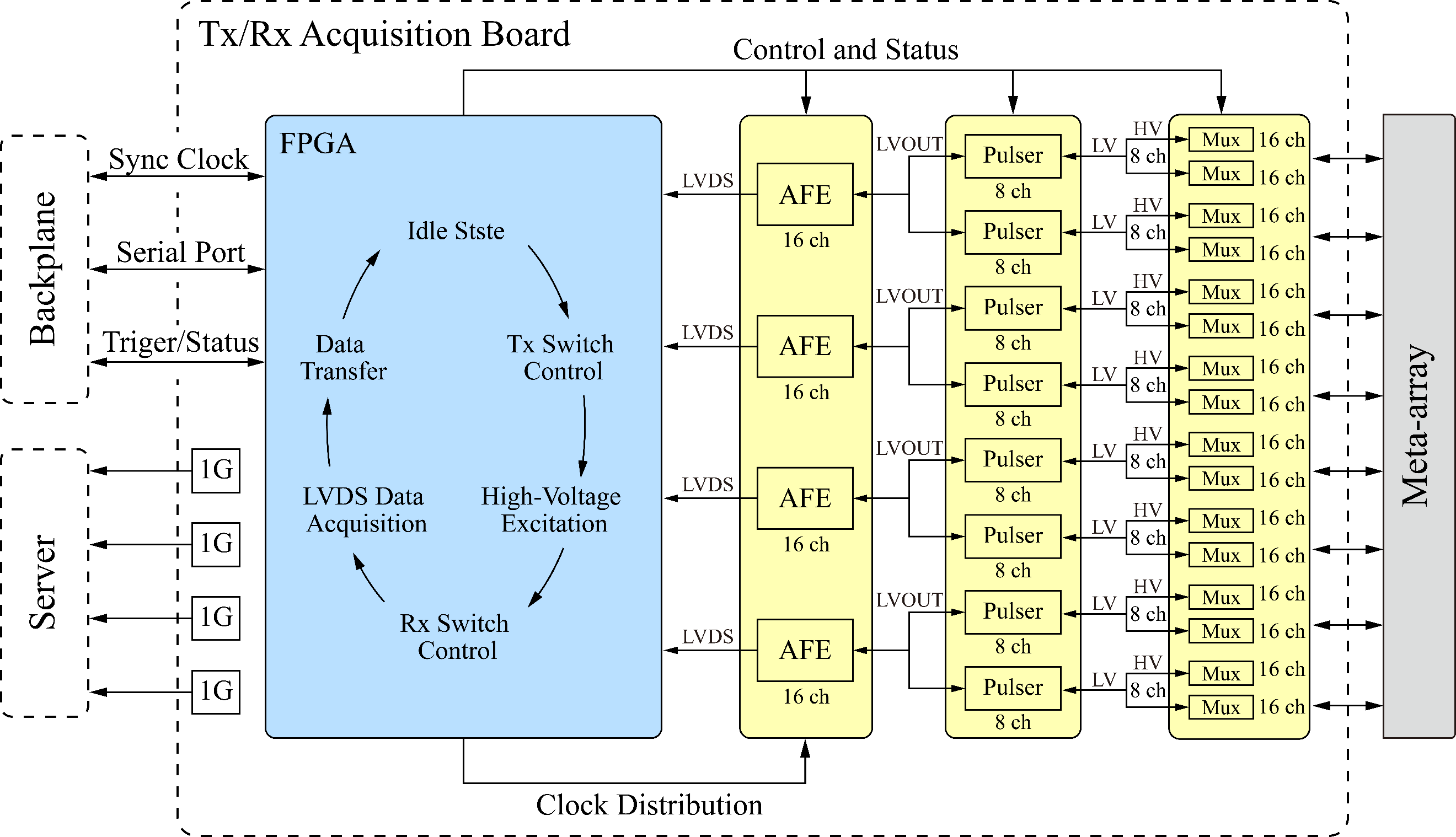


**Fig. S5. Architecture of the Tx/Rx acquisition board.**


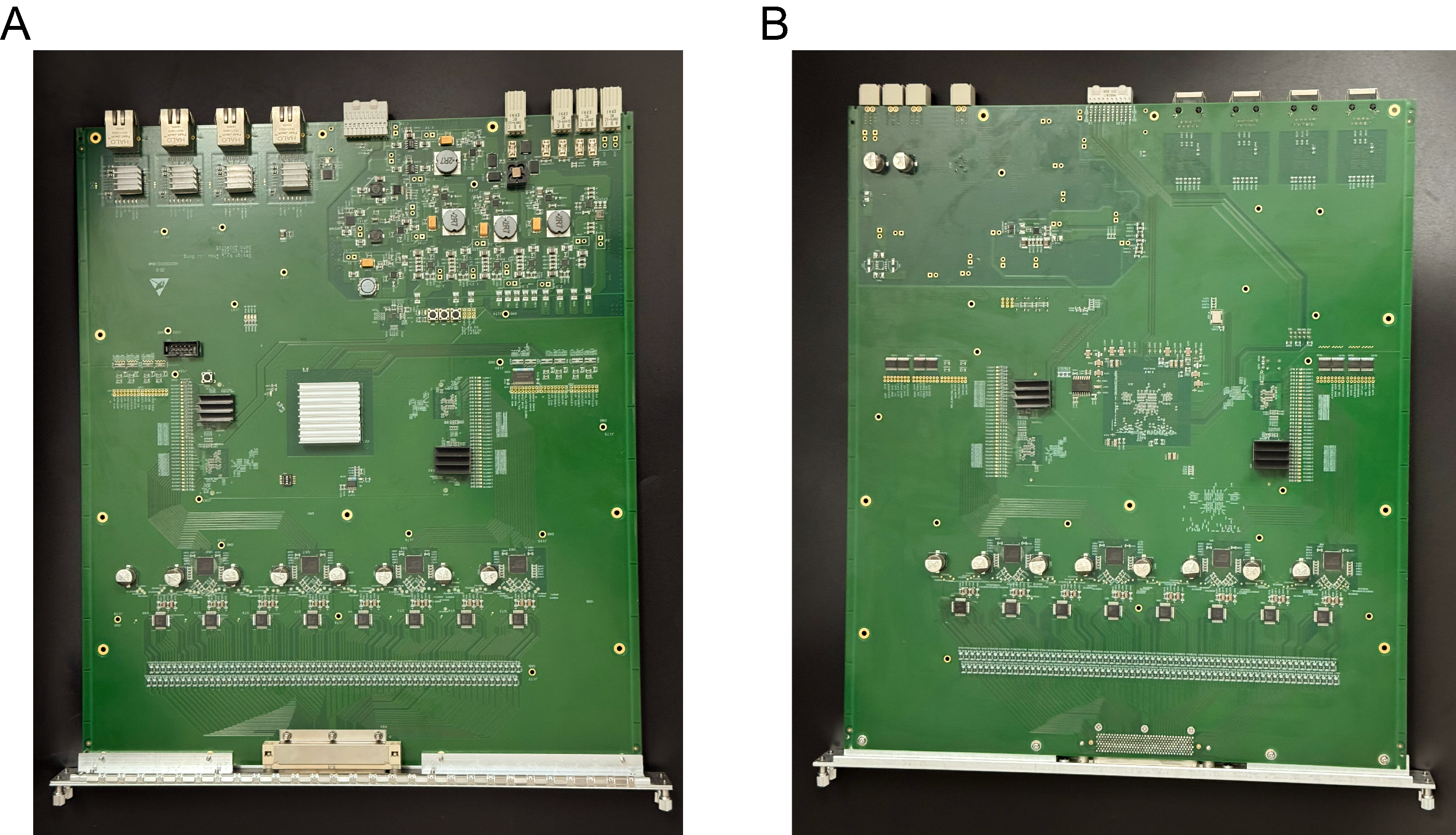


**Fig. S6. Photographs of the Tx/Rx acquisition board.** A, Front view. B, Rear view.

**
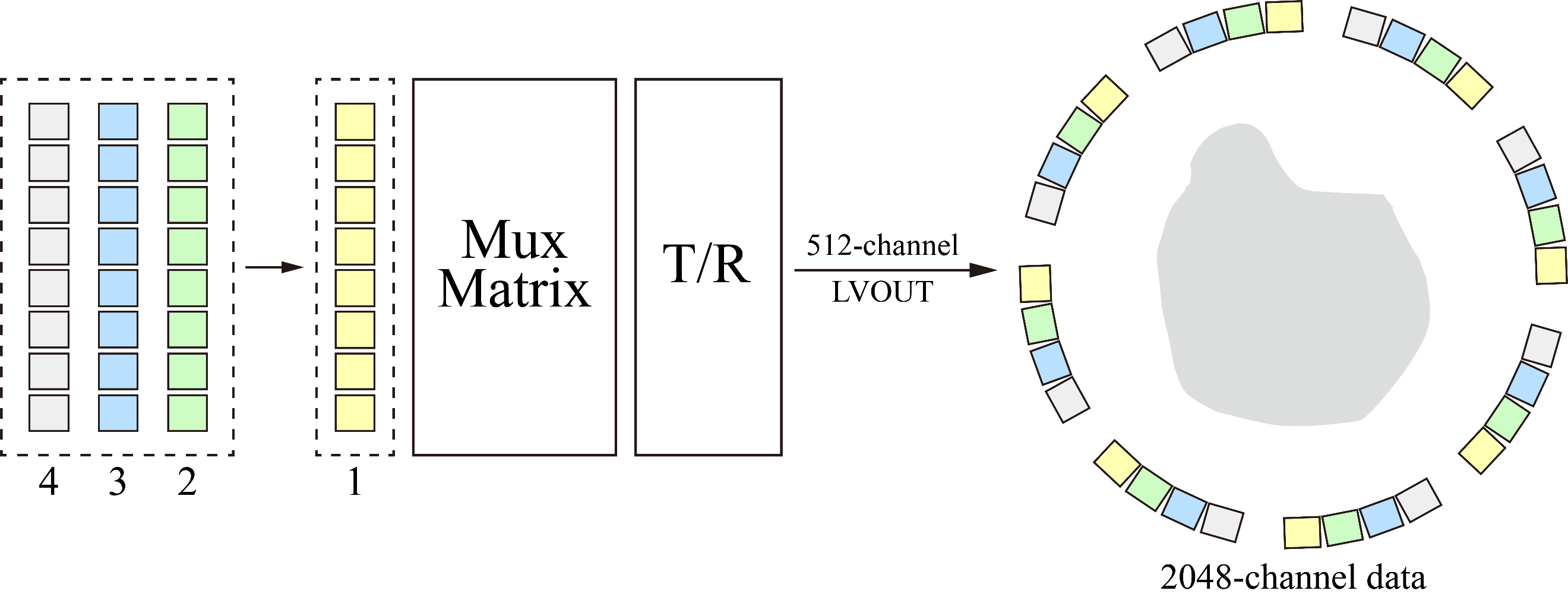
**

**Fig. S7. Time-multiplexed receive scheme**


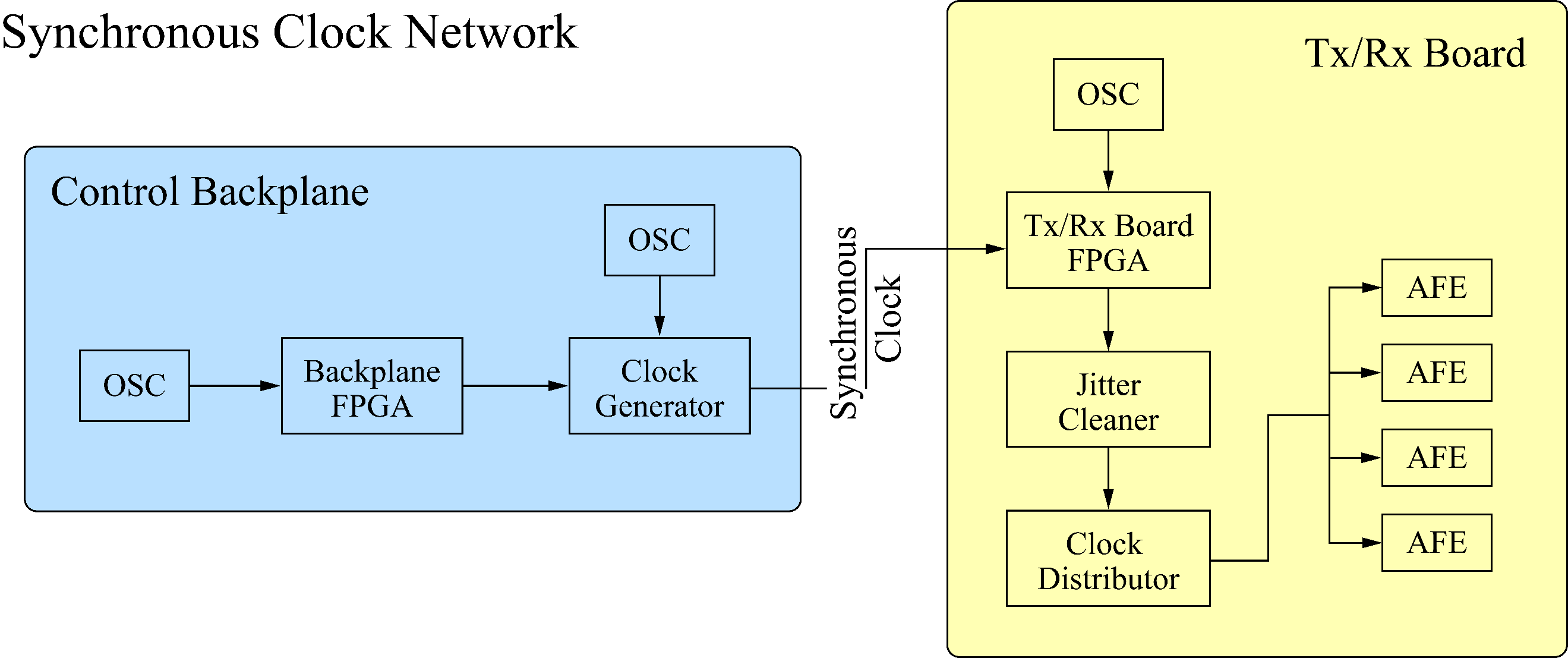


**Fig. S8. Architecture of the synchronous clock network.**

**
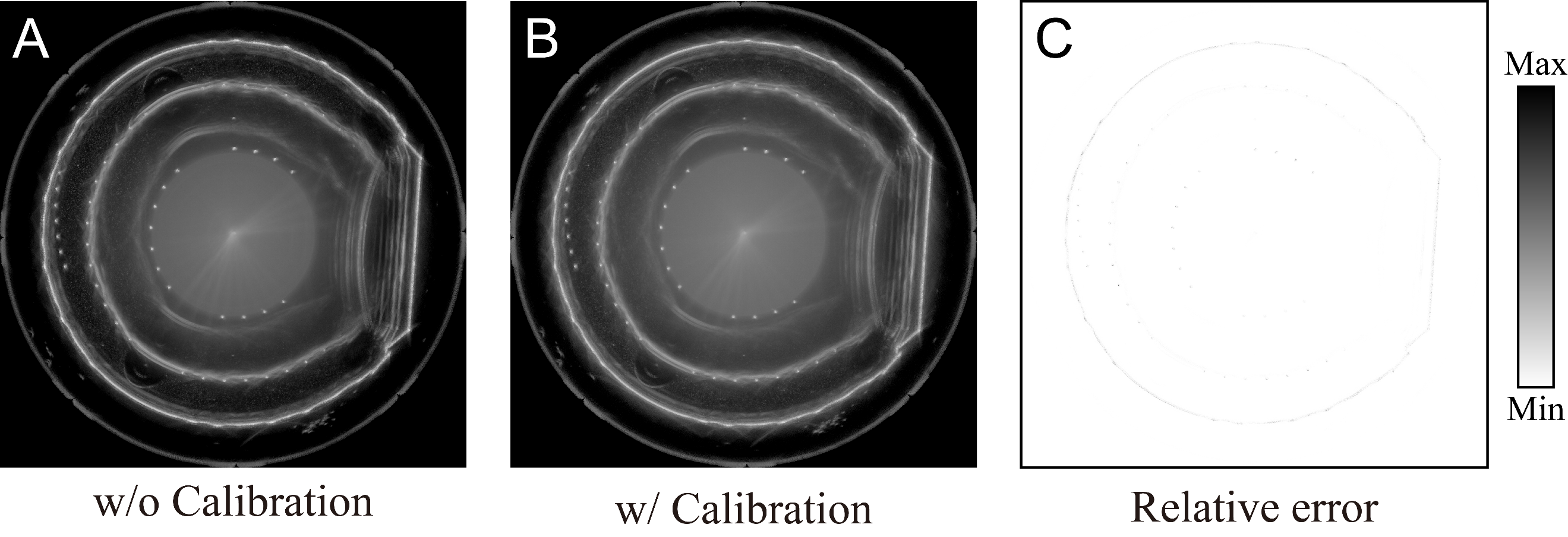
**

**Fig. S9. Impact of inter-array calibration on DMAS tomographic reconstruction.**

**
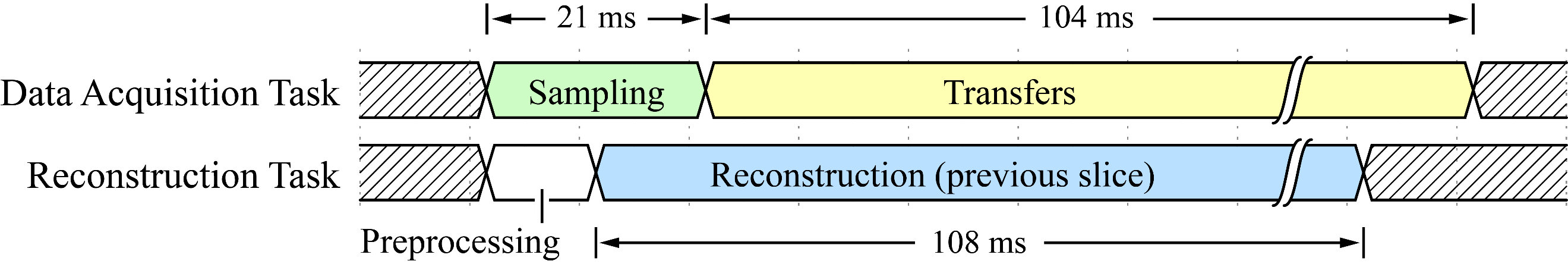
**

**Fig.S10. Timing diagram of pipeline tasks.**


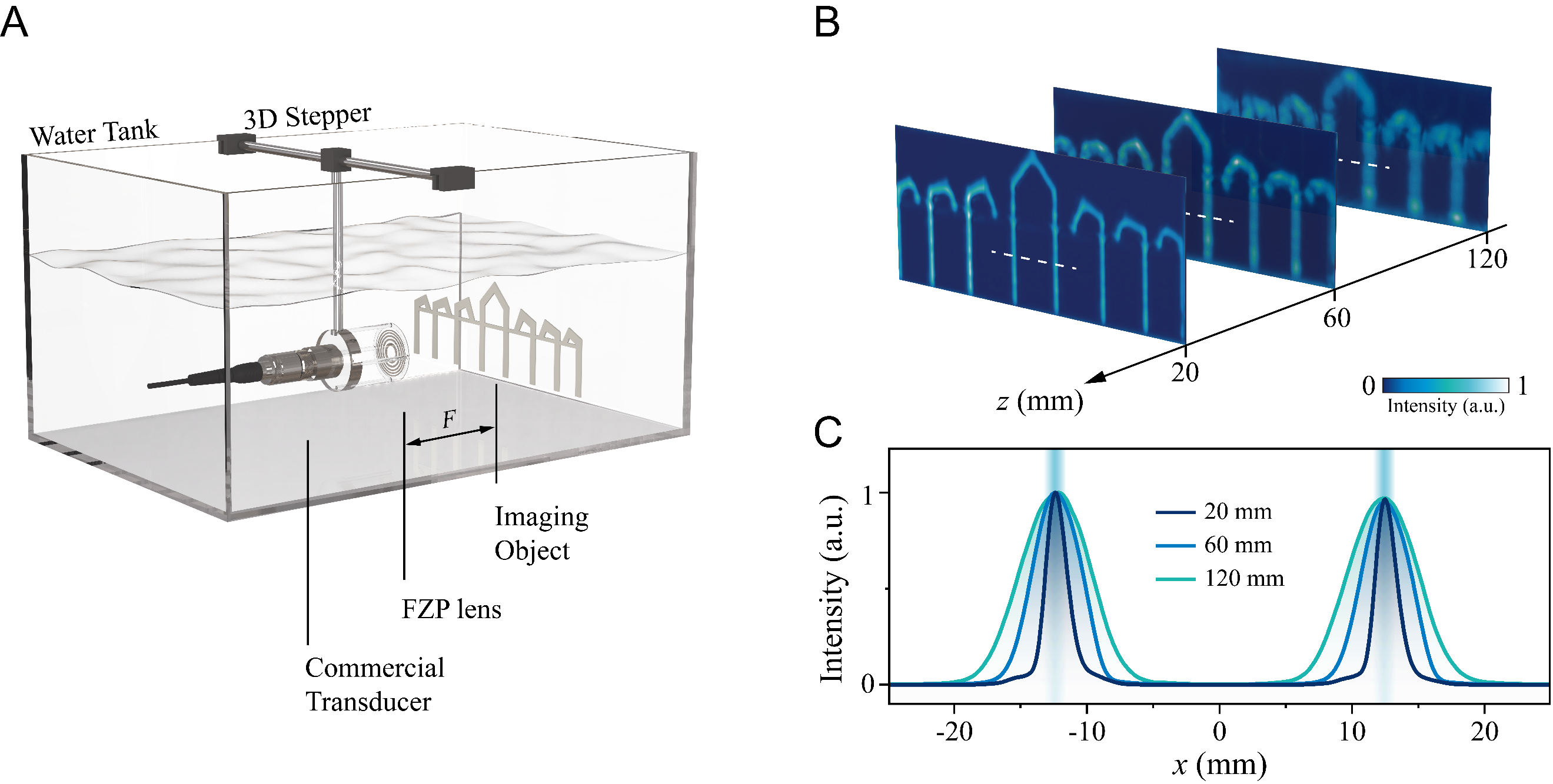


**Fig. S11. Echo imaging by using a FZP lens.** A, Experimental setups for the FZP-lens-based echo imaging. B, The ultrasonic images of the objects at different distances (20mm, 60mm, and 120mm) for testing lateral resolution. C, Intensity profiles extracted the dashed lines in B.


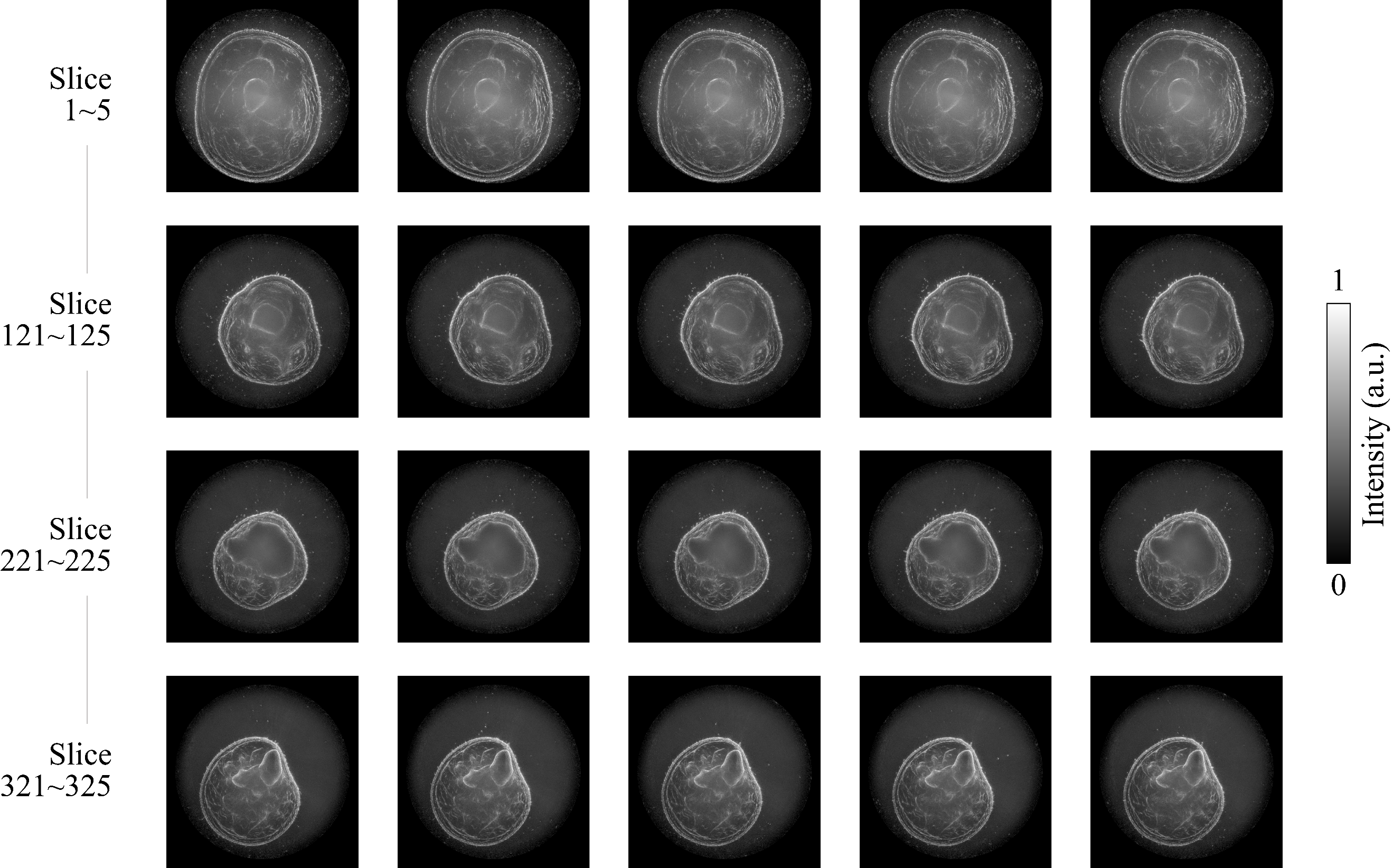


**Fig. S12. 2D high-resolution ultrasound images of sequential cross-sectional slices of the human leg.** The results are reconstructed from a total of 325 layers.


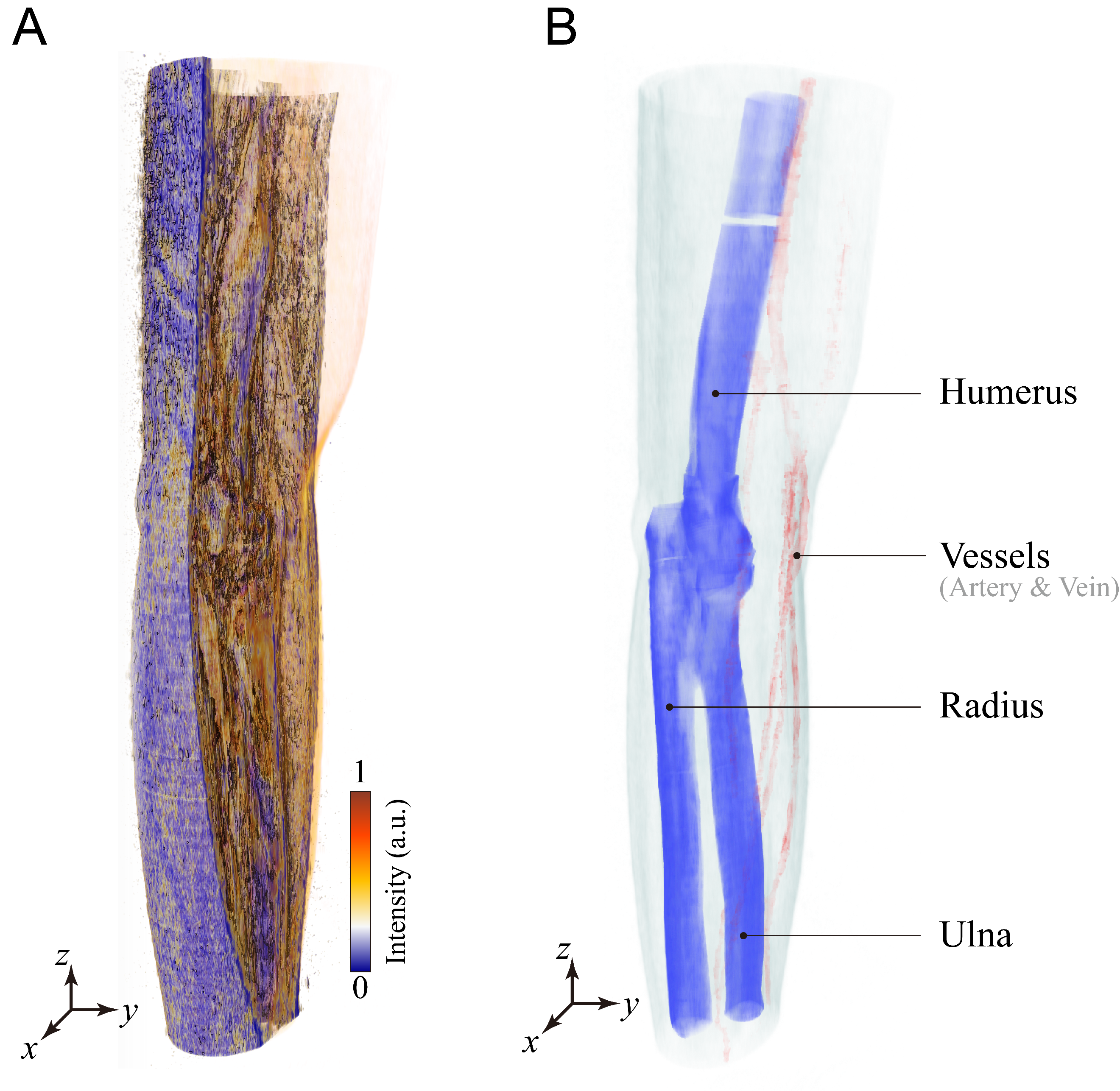


**Fig. S13. 3D tomographic imaging of human upper arm.** A, Tomography reconstructed from 325 consecutive high-resolution 2D slices. B, Segmentation results with the skeletal structures (humerus, radius and ulna) and vessels (artery and vein), shown in blue and red, respectively.

**
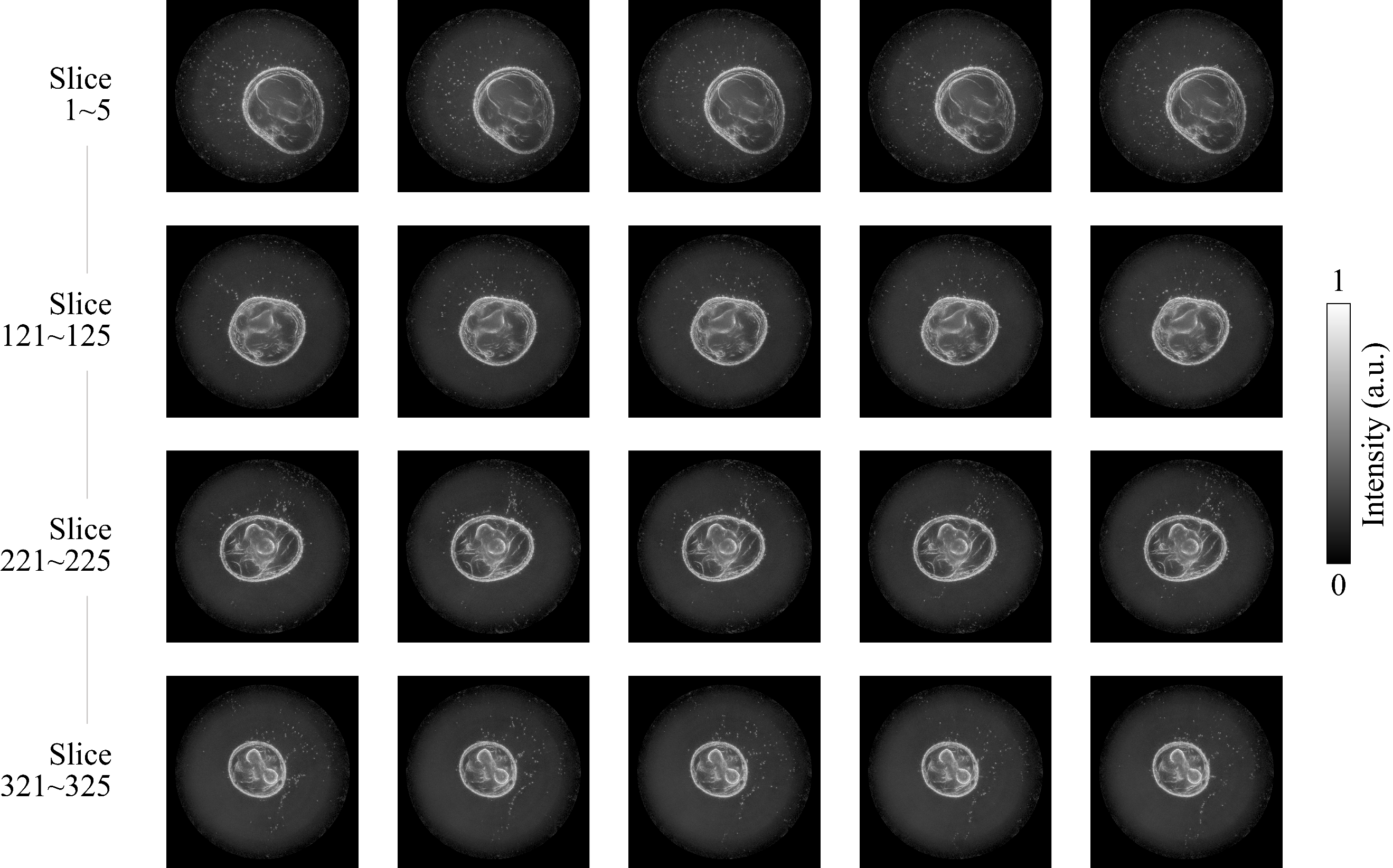
**

**Fig. S14. 2D high-resolution ultrasound images of sequential cross-sectional slices of the human upper arm.** The results are reconstructed from a total of 325 layers.

**
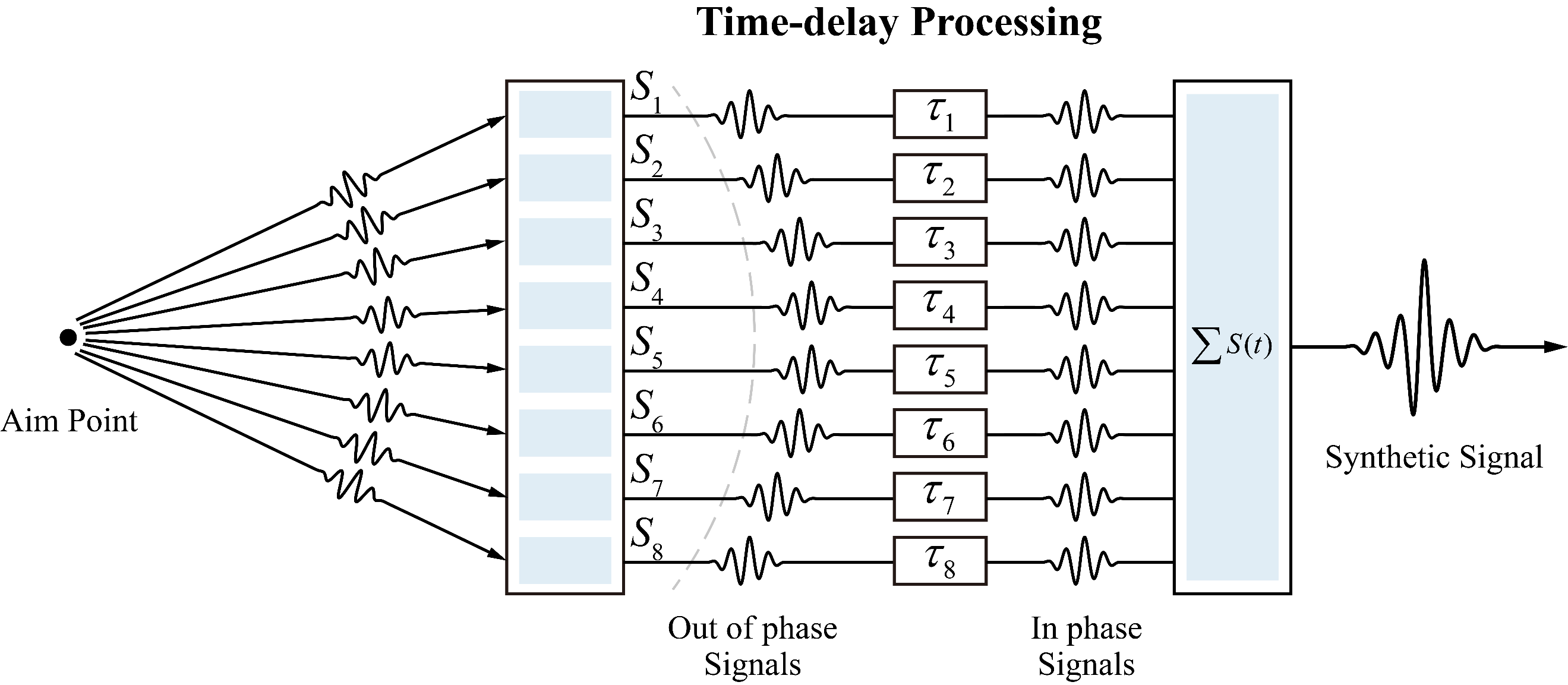
**

**Fig. S15. Schematic of delay-and-sum beamforming in receive mode.**


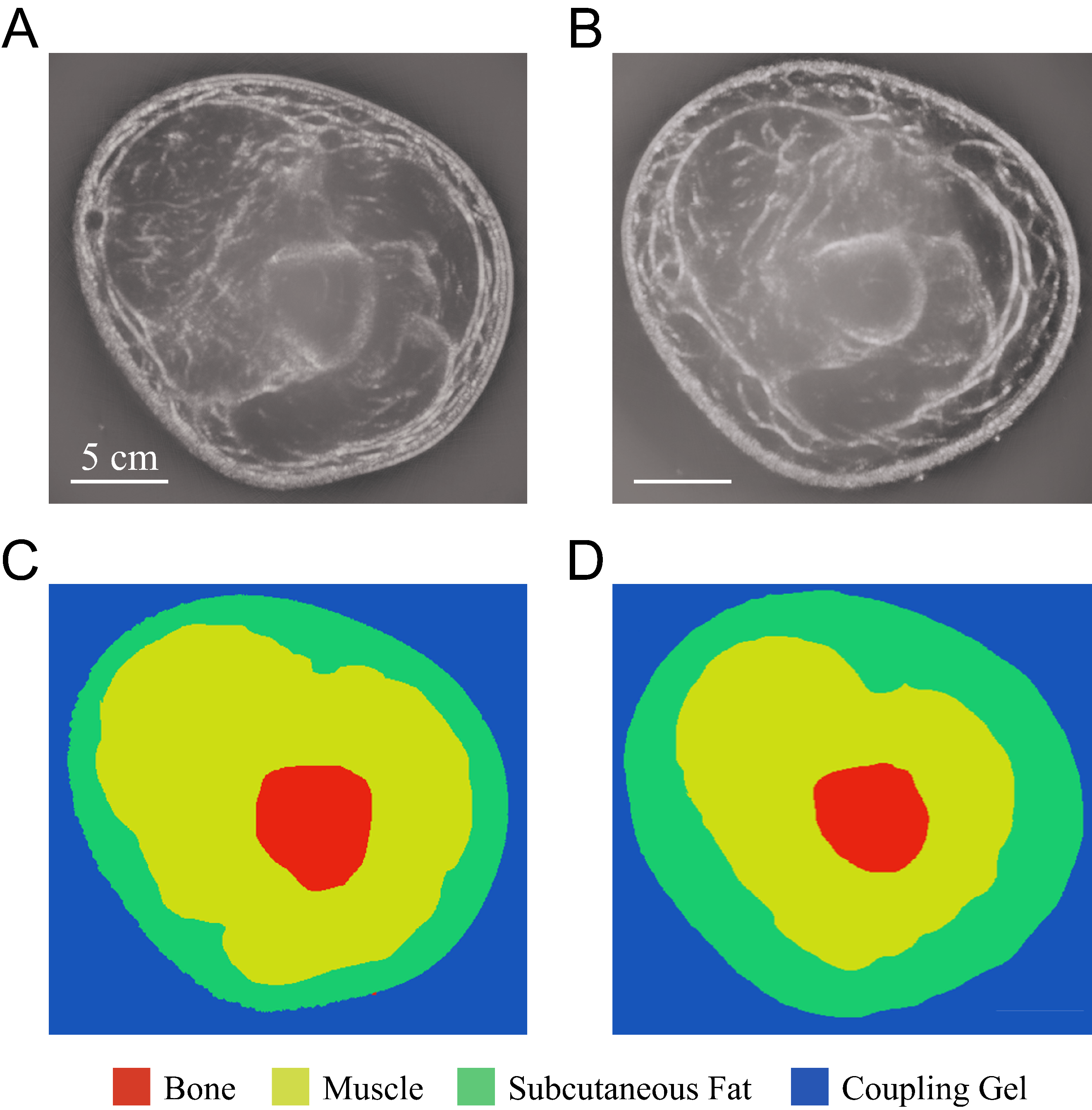


**Fig. S16. Cross-sectional comparison of the upper arm between an active and a sedentary 23-year-old male volunteer.** A-B. USCT slices of upper arm from the active volunteer (A) and the sedentary volunteer (B). C-D. Tissue segmentations of the upper arm, with bond in red, muscle in yellow, subcutaneous fat in green, and coupling gel in blue, shown for volunteer (A) and volunteer (B), respectively.

**Table S1.**

**The detailed acoustic parameters of the TM phantom.**

| **Component** | **Parameter** | **Specification** |
| --- | --- | --- |
| TM Phantom | Sound speed | 1540 ± 10m/s |
|  | Attenuation coefficient slope | 0.70 ± 0.05dB/cm/MHz |
| Nylon Wire | Sound speed | 2600 ± 80m/s |
|  | Diameter | 0.30 ± 0.05mm |

**Table S2.**

**Comparative analysis of cross-sectional areas of upper-arm tissue in two volunteers.**

| **Objects** | **Area (mm^2^)** | | | | **Muscle proportion** |
| --- | --- | --- | --- | --- | --- |
|  | **Upper-arm CS** | **Muscle** | **Bone** | **Subcutaneous fat** |  |
| Volunteer A | 5922.06 | 3611.01 | 495.32 | 1815.73 | 60.98% |
| Volunteer B | 6245.22 | 2880.18 | 411.22 | 2953.82 | 46.12% |
